# Avidity-Based Method for the Efficient Generation of Monoubiquitinated Recombinant Proteins

**DOI:** 10.1021/jacs.3c01943

**Published:** 2023-04-03

**Authors:** Spencer L. Nelson, Yunan Li, Yue Chen, Lalit Deshmukh

**Affiliations:** † Department of Chemistry and Biochemistry, University of California San Diego, La Jolla, California 92093, United States; ‡ Department of Biochemistry, Molecular Biology, and Biophysics, University of Minnesota at Twin Cities, Minneapolis, Minnesota 55455, United States

## Abstract

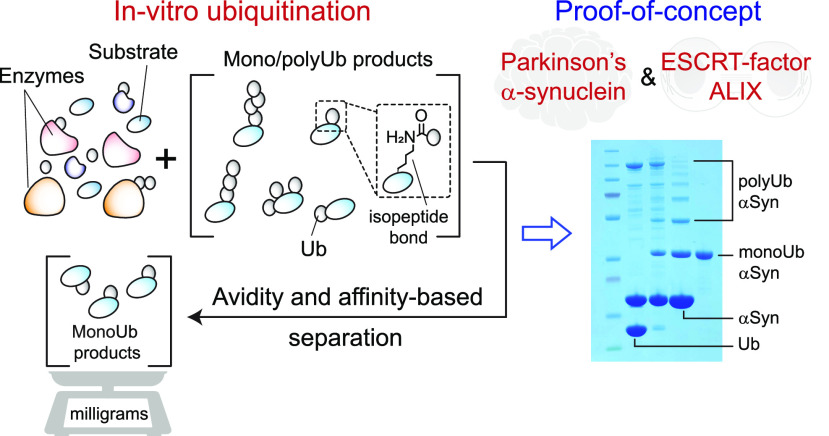

Monoubiquitination of proteins governs diverse physiological processes, and its dysregulation is implicated in multiple pathologies. The difficulty of preparing sufficient material often complicates the biophysical studies of monoubiquitinated recombinant proteins. Here we describe a robust avidity-based method that overcomes this problem. As a proof-of-concept, we produced milligram quantities of two monoubiquitinated targets, Parkinson’s protein α-synuclein and ESCRT-protein ALIX, using NEDD4-family E3 ligases. Monoubiquitination hotspots were identified by quantitative chemical proteomics. Using FRAP and dye-binding assays, we uncovered strikingly opposite effects of monoubiquitination on the phase separation and fibrillization properties of these two amyloidogenic proteins, reflecting differences in their intermolecular interactions, thereby providing unique insights into the impact of monoubiquitination on protein aggregation.

Protein ubiquitination orchestrates nearly all eukaryotic cellular events.^1^ It starts by attaching ubiquitin through isopeptide bonds to a single or multiple lysine residues of a target protein via a coordinated enzymatic reaction involving activating (E1), conjugating (E2), and ligating (E3) enzymes to form mono/multi-monoubiquitinated products. Further modification of ubiquitin’s seven lysine residues and its N-terminal methionine creates moieties decorated with polyubiquitin chains. These post-translational modifications (mono-, multimono-, and polyubiquitination) encode specific signals that are decoded by deubiquitinating enzymes and proteins containing ubiquitin-binding domains. Among these, monoubiquitination is the most prevalent,^2^ and is involved in various physiological processes (e.g., chromatin regulation, DNA damage response, protein sorting, trafficking, and degradation), viral egress, genetic disorders, and neurodegenerative proteinopathies.^3^ Although the mechanisms that restrict the substrates to monoubiquitination, preventing polyubiquitination, are not clearly understood, monoubiquitinated proteins are often modified at multiple individual sites, creating a pool of heterogeneous populations.^4,5^ The frequency with which each site gets ubiquitinated and the collective effects of these modifications on the physicochemical characteristics of the target protein are usually unclear since obtaining such samples in sufficiently high yields and purities for biophysical studies is challenging. This is because enzymatic reactions performed on recombinant substrates often generate a composite mixture containing reaction components and mono-, multimono-, and polyubiquitinated products, and selective purification of monoubiquitinated species from this soup is difficult. Additionally, chemical (nonenzymatic) methods that can produce isopeptide-linked monoubiquitinated proteins are technically challenging and not applicable to most proteins.^6^ There is, therefore, a need for a technique that can facilitate the high-yield production of monoubiquitinated proteins. Here we present an efficient approach that fills this gap.

This method can be applied to recombinant substrates with specific ubiquitinating enzymes. As a proof-of-concept, we used two substrates, α-synuclein and apoptosis-linked gene-2-interacting protein X (ALIX), and enzymes UbE1, UbE2D3, and neuronal precursor cell-expressed developmentally downregulated 4 (NEDD4)-family E3 ligases (NEDD4L and WW domain containing E3 ubiquitin protein ligase 2 (WWP2)); Figure 1A. Aberrant aggregation of α-synuclein is a hallmark of Parkinson’s disease.^7,8^ α-synuclein accumulated in the Lewy bodies of Parkinson’s patients is often mono- and diubiquitinated,^9^ perhaps due to the breakdown of its degradation pathways. Endosomal sorting complex required for transport (ESCRT)-protein ALIX governs multiple processes, including protein sorting, neurodevelopment, cytokinesis, and enveloped virus budding.^10−12^ Like many ESCRT-proteins, ALIX undergoes monoubiquitination in vivo.^13,14^ All nine members of the NEDD4-family ligases collaborate with UbE1 and UbE2D3 to promote the ubiquitination of cellular proteins.^8^ Specifically, NEDD4L ubiquitinates α-synuclein in the postischemic brain, promoting its degradation via the endolysosomal pathway,^15^ whereas NEDD4L and WWP2 are involved in ALIX’s monoubiquitination, vital for its roles in human immunodeficiency virus 1 (HIV-1) budding^16^ and lysosomal sorting of G protein coupled receptors (GPCRs).^17^ Although mono- and polyubiquitinated α-synuclein was produced using chemical methods,^18−20^ no such attempts were made for ALIX, due to the problems associated with its recombinant expression stemming from ribosomal stalling induced by its amyloidogenic proline-rich domain (PRD).^12,21^ We recently overcame these expression issues by introducing a P801G mutation in its PRD and established that ALIX phase separates via its PRD, crucial for its role in cytokinetic abscission (manuscript submitted). However, the effects of monoubiquitination on ALIX’s aggregation and functions are unclear.

**Figure 1:**
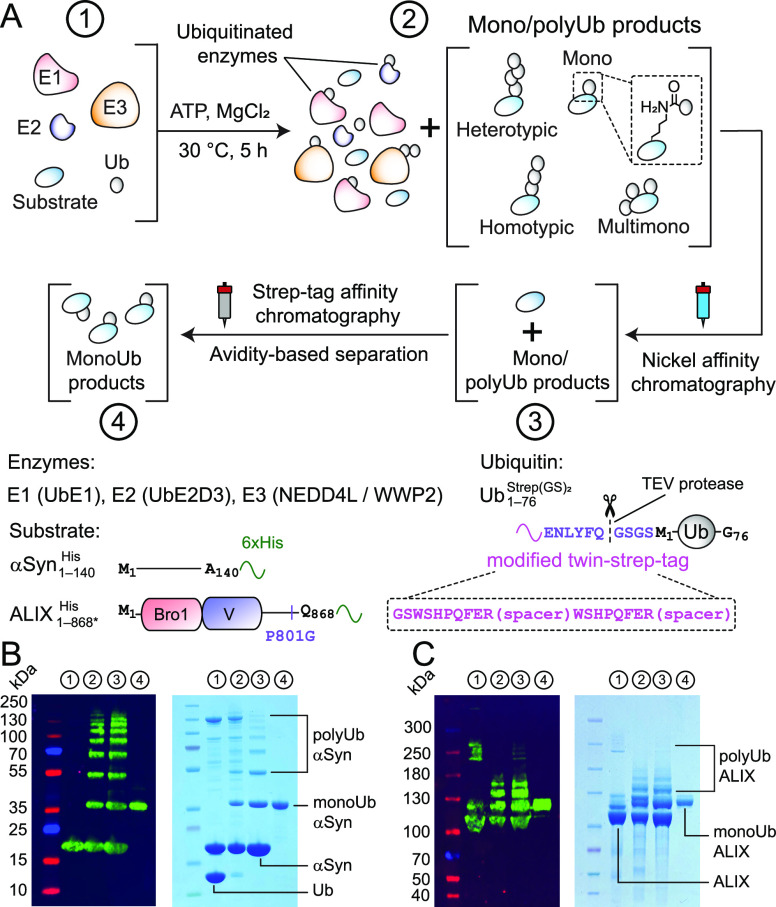
Large-scale production of monoubiquitinated proteins. (A) In vitro ubiquitination reaction (Step 1) to produce ubiquitinated products with native isopeptide linkages, highlighted in the dashed square (Step 2). The lower panel denotes the constructs that were used (Figure S1 and Table S1). The reaction components were subjected to affinity chromatography (Steps 3 and 4) for a selective purification of monoubiquitinated products. Western blot and SDS-PAGE analyses of corresponding reactions of (B) αSyn_1–140_^His^ + NEDD4L and (C) ALIX_1–868*_^His^ + WWP2; 4–12% Bis-Tris and 3–8% Tris-Acetate gels were used for αSyn_1–140_^His^ and ALIX_1–868*_^His^, respectively. Aliquots from each step are designated by a circled number.

All recombinant enzymes were expressed with N-terminal polyhistidine affinity tags, which were cleaved using tobacco etch virus (TEV)-protease during the final stages of purification. α-synuclein and ALIX were expressed with noncleavable C-terminal polyhistidine tags, αSyn_1–140_^His^ and ALIX_1–868*_^His^ (the asterisk denotes P801G mutation), respectively; Figure 1A. The ubiquitin construct carried a modified N-terminal twin-strep tag^22^ and a TEV-protease cleavage site, Ub_1–762_^Strep(GS)_2_^; see Figure S1 for the rationale used for the design of this tag and Figures S2 and S3 for the nuclear magnetic resonance (NMR) analyses of Ub_1–762_^Strep(GS)_2_^, which revealed a minimal impact of the tag on ubiquitin’s structure. The enzymes and Ub_1–762_^Strep(GS)_2_^ were mixed with substrates (αSyn_1–140_^His^/ALIX_1–868*_^His^) and incubated with ATP and MgCl_2_ to generate mono-, multimono-, and polyubiquitinated products. Nickel affinity chromatography facilitated a selective purification of substrate and its ubiquitinated products. Monoubiquitinated species were separated from this mixture using strep-tag affinity chromatography by exploiting the avidity effect.^23^ This is because unlike monoubiquitinated products, multimono-/polyubiquitinated moieties bound extremely tightly to the resin-coupled strep-tactin, a derivative of tetrameric streptavidin, and therefore, could not be readily displaced by the competitive binding reagent, biotin (Figures S4 and S5). Western blot and sodium dodecyl-sulfate polyacrylamide gel electrophoresis (SDS-PAGE) analyses of αSyn_1–140_^His^ + NEDD4L and ALIX_1–868*_^His^ + WWP2 reactions and purification of monoubiquitinated products are shown in Figure 1B–C. Both reactions generated milligram quantities of monoubiquitinated products (Figures S6 and S7 and Table S2), attesting to the efficacy of this method.

The high purity of the monoubiquitinated α-synuclein facilitated a detailed stoichiometric analysis using our chemical proteomics approach (Figure 2A–C).^24^ Here, unmodified lysine residues of a target protein are conjugated to an acetyl-GG-*N*-Hydroxysuccinimide (NHS) tag, followed by proteolytic digestion and secondary labeling with ^13^C-acetyl-NHS, thereby generating fragments of the originally ubiquitinated peptides and their unmodified counterparts that are structurally identical but differ in ^13^C-labeling (Figure 2B). Subsequent liquid chromatography-mass spectrometry (LC-MS) analysis of these fragments allows quantification of site-specific monoubiquitination frequency via a comparison of the corresponding chromatography peak-area ratios (Figure 2C and Table S3). The N-terminal membrane binding region (MBR), central nonamyloid component (NAC), and C-terminal region (CTR) of α-synuclein were monoubiquitinated by NEDD4L at 37%, 21%, and 42%, respectively. The collective high-frequency (63%) of monoubiquitination of residues in the NAC (K80) and CTR (K96/K97/K102) of α-synuclein is consistent with the fact that NEDD4-ligases bind to the proline-rich region of its CTR.^8,25^ Although the stoichiometric deconvolution of immediately adjacent lysine residues (e.g., K96/K97/K102 of the CTR) was not feasible, we were able to quantify monoubiquitination frequencies for sufficiently distant residues of the MBR (e.g., 19% monoubiquitination at K6/K10/K12 vs 11% at K21/K23). A similar analysis of multimono/polyubiquitinated α-synuclein produced in these ubiquitination reactions revealed changes in enzymatic preferences upon progressive addition of ubiquitin moieties (Figure S4B and Table S4). Similar quantification of cerebral α-synuclein is difficult owing to endogenous deubiquitinating enzymes and the rapid deubiquitination in post-mortem samples.^25^ Hence, the above approach identifies ubiquitination hotspots for a given group of enzymes and their substrate and provides important insights regarding the corresponding in vivo ubiquitination pattern for the said group.

**Figure 2:**
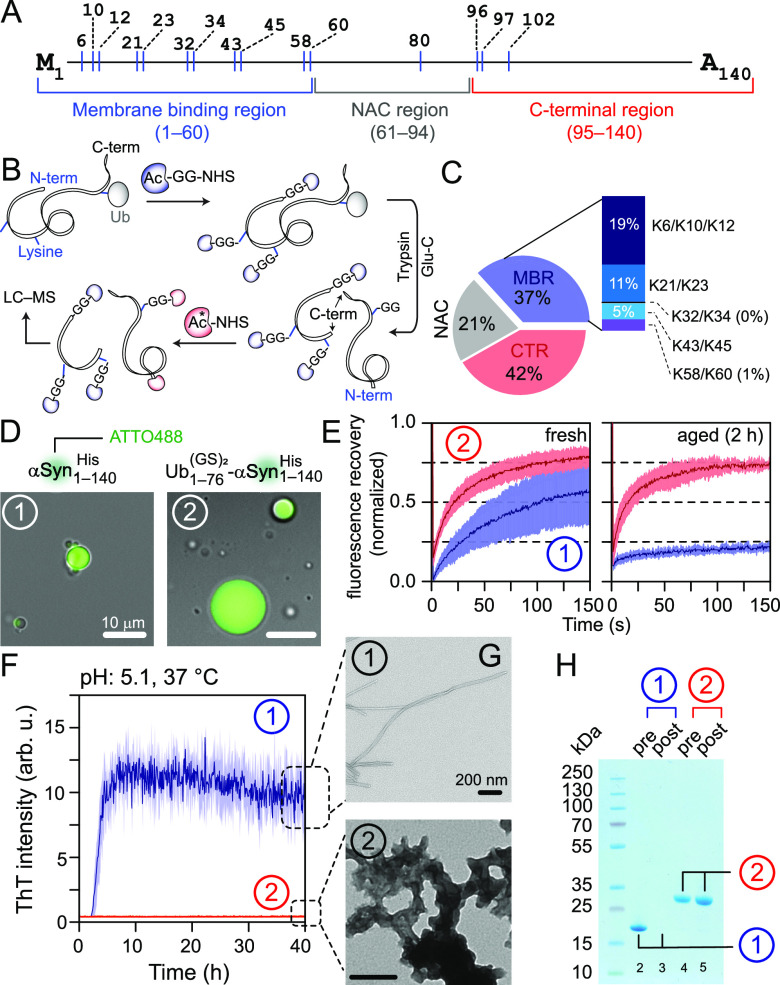
Impact of NEDD4L-mediated monoubiquitination on α-synuclein’s aggregation. Schemes of (A) α-synuclein and (B) quantitative chemical proteomics used to determine monoubiquitination stoichiometry; the asterisk denotes ^13^C-labeled acetyl-NHS. (C) Pie-chart of the average frequency of monoubiquitination in the MBR (blue), NAC (gray) and CTR (red) regions of αSyn_1–140_^His^ (*n* = 2). Unlike the CTR, the monoubiquitination frequency for the individual lysine residues of MBR could be deconvoluted, represented by a stacked bar. (D) Microscopy images of droplets of αSyn_1–140_^His^ and its monoubiquitinated counterpart with 10% w/v PEG-8000, represented by circled no. 1 and 2, respectively; the same numbering scheme is used in the remaining panels. (E) FRAP analysis of freshly prepared and aged condensates with the solid line and shaded region representing the mean and SD (*n* = 3), and blue and red colors for unmodified and monoubiquitinated αSyn_1–140_^His^, respectively. (F) Aggregation of non- and monoubiquitinated αSyn_1–140_^His^ studied by ThT assays (*n* = 2); the same color-scheme as E. (G) Negative-stain EM images of aggregated samples from F showing fibrils for αSyn_1–140_^His^ and amorphous aggregates for its monoubiquitinated moieties. (H) SDS-PAGE analysis of pre- and postaggregated samples from F. The lack of band intensity in lane-3 is due to αSyn_1–140_^His^ fibrillization.

Both unmodified and monoubiquitinated α-synuclein phase separated into condensates with a molecular crowder polyethylene glycol (PEG)-8000, Figures 2D and S8–S9. Although freshly made condensates of both moieties were dynamic, as evidenced by fluorescence recovery after photobleaching (FRAP) assays (Figure 2E), condensates of α-synuclein exhibited a noticeably lower fluorescence recovery than those of its monoubiquitinated counterpart (60% vs 80% average recovery in 150 s, respectively). Moreover, the fluorescence recovery of α-synuclein condensates decreased significantly after a 2 h incubation at room temperature, whereas the condensates of monoubiquitinated α-synuclein remained dynamic (20% vs 75% recovery, respectively). The latter frequently coalesced and increased significantly in size with time (Video S1). These observations indicate a time-dependent gelation of α-synuclein droplets, perhaps due to its fibrillization and the lack thereof for its monoubiquitinated moieties. Aggregation assays performed using an amyloid-sensitive dye, Thioflavin T (ThT), confirmed this hypothesis, with sigmoidal aggregation profiles for α-synuclein, a hallmark of fibrillization, and no noticeable increase in ThT signals for its monoubiquitinated counterpart (Figure 2F). Negative-stain electron microscopy (EM) and SDS-PAGE analyses demonstrated the presence of SDS-resistant fibrils and nonfibrillar aggregates for unmodified and monoubiquitinated α-synuclein, respectively (Figure 2G–H). These results demonstrate that NEDD4L-mediated monoubiquitination of α-synuclein creates dynamic condensates and makes it resistant to fibrillization. This is likely due to modifications of lysine residues in and around its aggregation-prone NAC region,^26,27^ thereby preventing fibrillization through modulation of intermolecular interactions.

Solution NMR analysis established that the ALIX-V domain binds to ubiquitin, Figures 3A–B and S10, consistent with a prior report that measured a dissociation constant of ∼120 μM for this interaction.^28^ Analysis of monoubiquitinated ALIX_1–868*_^His^ using quantitative chemical proteomics showed that the V-domain is significantly more monoubiquitinated by NEDD4L and WWP2 (72% and 60% monoubiquitination, respectively, Figure 3C and Table S3) than Bro1 and PRD of ALIX. Examination of site-specific frequencies revealed two significant differences (Figure 3D). Residues K501/K510 of V-domain were monoubiquitinated at 44% vs 9% while residue K420 was monoubiquitinated at 8% vs 24% by NEDD4L and WWP2, respectively, highlighting the site-specific preferences of these two ligases. ALIX and its monoubiquitinated counterpart formed gel-like condensates with PEG-4000, as evidenced by fluorescence microscopy and negligible FRAP recoveries (Figures 3E–F and S8 and S9), consistent with our recent findings that ALIX makes nondynamic condensates that confine abscission factors (manuscript submitted). Unlike α-synuclein (cf. Figure 2E), monoubiquitinated ALIX did not form dynamic condensates (Figure 3F and S12A), possibly because monoubiquitinated ALIX molecules bound to one another via their V-domains, thereby creating optimal conditions for nucleation and growth of ALIX fibrils. ThT assays, negative-stain EM, and SDS-PAGE analyses confirmed this hypothesis and revealed that, unlike unmodified ALIX, its monoubiquitinated counterpart formed amyloid fibrils (Figures 3G–I and S12B). Such fibrils in vivo will likely act as a scaffolding platform, aiding the formation of downstream ESCRT filaments needed for membrane scission, thereby facilitating ALIX’s versatile functions.

**Figure 3:**
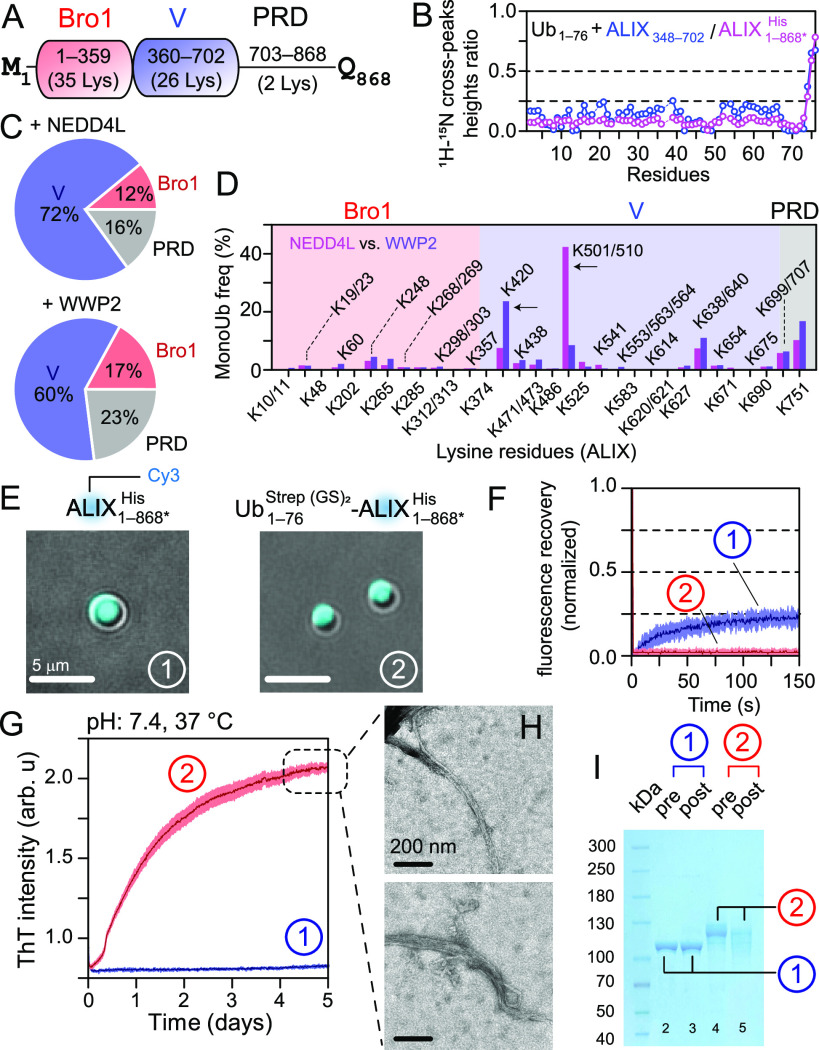
NEDD4L/WWP2-mediated monoubiquitination of ALIX. (A) Scheme of ALIX (also see Figure S11). (B) The reduction in ^1^H_N_/^15^N cross-peak heights of 100 μM ^15^N-labeled ubiquitin with 100 μM nonlabeled ALIX_348–702_ (blue) and ALIX_1–868*_^His^ (pink). (C) Pie-charts illustrating the average frequency of monoubiquitination in individual ALIX domains using NEDD4L (upper) and WWP2 (lower); *n* = 2. (D) Bar-chart of site-specific differences in monoubiquitination frequencies of ALIX residues brought out by NEDD4L (pink) and WWP2 (magenta); arrows mark significant differences. Only ubiquitinated residues are plotted. (E) Microscopy images of condensates of ALIX_1–868*_^His^ and its WWP2-mediated monoubiquitinated counterpart (5% w/v PEG-4000), represented by circled no. 1 and 2; the same numbering scheme is used in the remaining panels. (F) Poor FRAP recoveries (<25%) for the freshly prepared condensates of ALIX_1–868*_^His^ (blue) and its WWP2-mediated monoubiquitinated moieties (red), *n* = 3, the same coloring scheme in the remaining panels. (G) Fibrillization of monoubiquitinated ALIX_1–868*_^His^ and the lack thereof for its nonubiquitinated species by ThT assays, *n* = 2. (H) Negative-stain EM analyses of fibrils formed by monoubiquitinated ALIX_1–868*_^His^. (I) SDS-PAGE analysis of pre- and postaggregated samples from G. Fibrillization of monoubiquitinated ALIX resulted in a decreased band intensity in lane-5 as compared to the preaggregated sample in lane-4.

In summary, we devised an efficient method to purify milligram quantities of monoubiquitinated proteins. It facilitated detailed analyses of ubiquitination hotspots and the impact of monoubiquitination on α-synuclein and ALIX aggregation. This study utilized recombinant NEDD4-E3 ligases, which ubiquitinate targets at multiple sites. Given its ease-of-use, this method will apply to similar systems, including ligases that modify a specific lysine, and lays a solid foundation for our ongoing efforts to produce polyubiquitinated recombinant proteins. Additionally, it will serve as a template to generate small ubiquitin-related modifier (SUMO)-ylated proteins,^29^ a posttranslational modification analogous to ubiquitination.

## Data Availability

Human UbE1 (UniProt accession no. P22314), UbE2D3 (UniProt accession no. P61077), NEDD4L (UniProt accession no. Q96PU5), WWP2 (UniProt accession no. O00308), ubiquitin (UniProt accession no. P0CG48), α-synuclein (UniProt accession no. P37840), and ALIX (UniProt accession no. Q8WUM4). The constructs of Ub_1–762_^Strep(GS)_2_^, UbE1, UbE2D3, NEDD4L, WWP2, ALIX_1–868*_^His^, ALIX_348–702_, and αSyn_1–140_^His^ have been deposited in the Addgene repository as accession numbers 186803, 186804, 186805, 186806, 186807, 186808, 189819, and 186802, respectively. The NMR chemical shift assignments of Ub_1–762_^Strep(GS)_2_^ have been deposited in the Biological Magnetic Resonance Bank as entry 51647. The mass spectrometry proteomics data have been deposited to the ProteomeXchange Consortium via the PRIDE partner repository as entry PXD037416.

## Supporting Information Available

Experimental details, recombinant constructs used in current study, NMR chemical shift analysis of ubiquitin constructs used in current study, Backbone RDC analysis of Ub_1–76_^Strep(GS)_2_^, exploiting the avidity effect for the selective purification of monoubiquitinated species, Strep-tag affinity chromatography for a selective purification of monoubiquitinated moieties, mass spectrometry analysis of monoubiquitinated α-synuclein and ALIX, solution NMR analysis of monoubiquitinated α-synuclein, phase separation of α-synuclein and ALIX and the impact of monoubiquitination, panoramic images showing the phase separation of proteins used in current study, NMR analyses of ubiquitin–ALIX interactions, ALIX and the location of its lysine residues, aggregation properties of NEDD4L-mediated monoubiquitinated ALIX, recombinant constructs used in current study, components of in vitro ubiquitination reactions and the yields of corresponding monoubiquitinated products, quantification of the site-specific frequency of monoubiquitination αSyn_1–140_^His^ and ALIX_1–868*_^His^ using a chemical proteomics approach, and quantification of the site-specific frequency of multi-mono/polyubiquitinated αSyn_1–140_^His^ using chemical proteomics approach (PDF)
Video S1: Droplet fusion of unmodified and monoubiquitinated α-synuclein (AVI)

## Supplementary Material

ja3c01943_si_001.pdf

ja3c01943_si_002.avi

## References

[ref1] Swatek K. N.; Komander D. Ubiquitin modifications. Cell Res. 2016, 26 (4), 399–422. 10.1038/cr.2016.39. 27012465 PMC4822133

[ref2] Kaiser S. E.; Riley B. E.; Shaler T. A.; Trevino R. S.; Becker C. H.; Schulman H.; Kopito R. R. Protein standard absolute quantification (PSAQ) method for the measurement of cellular ubiquitin pools. Nat. Methods 2011, 8 (8), 691–696. 10.1038/nmeth.1649. 21743460 PMC3196335

[ref3] Chen Y.; Zhou D.; Yao Y.; Sun Y.; Yao F.; Ma L. Monoubiquitination in homeostasis and cancer. Int. J. Mol. Sci. 2022, 23 (11), 5925 10.3390/ijms23115925. 35682605 PMC9180643

[ref4] Rott R.; Szargel R.; Haskin J.; Shani V.; Shainskaya A.; Manov I.; Liani E.; Avraham E.; Engelender S. Monoubiquitylation of alpha-synuclein by seven in absentia homolog (SIAH) promotes its aggregation in dopaminergic cells. J. Biol. Chem. 2008, 283 (6), 3316–3328. 10.1074/jbc.M704809200. 18070888

[ref5] Nakagawa T.; Nakayama K. Protein monoubiquitylation: targets and diverse functions. Genes Cells 2015, 20 (7), 543–62. 10.1111/gtc.12250. 26085183 PMC4744734

[ref6] Stanley M.; Virdee S. Chemical ubiquitination for decrypting a cellular code. Biochem. J. 2016, 473 (10), 1297–314. 10.1042/BJ20151195. 27208213 PMC5298413

[ref7] Stefanis L. α-Synuclein in Parkinson’s disease. Cold Spring Harb. Perspect. Med. 2012, 2 (2), a009399 10.1101/cshperspect.a009399. 22355802 PMC3281589

[ref8] Conway J. A.; Kinsman G.; Kramer E. R. The role of NEDD4 E3 ubiquitin–protein ligases in Parkinson’s disease. Genes 2022, 13 (3), 513 10.3390/genes13030513. 35328067 PMC8950476

[ref9] Tofaris G. K.; Razzaq A.; Ghetti B.; Lilley K. S.; Spillantini M. G. Ubiquitination of α-synuclein in Lewy bodies is a pathological event not associated with impairment of proteasome function. J. Biol. Chem. 2003, 278 (45), 44405–11. 10.1074/jbc.M308041200. 12923179

[ref10] Odorizzi G. The multiple personalities of ALIX. J. Cell Sci. 2006, 119 (15), 3025–3032. 10.1242/jcs.03072. 16868030

[ref11] Laporte M. H.; Chatellard C.; Vauchez V.; Hemming F. J.; Deloulme J.-C.; Vossier F.; Blot B.; Fraboulet S.; Sadoul R. ALIX is required during development for normal growth of the mouse brain. Sci. Rep. 2017, 7 (1), 44767 10.1038/srep44767. 28322231 PMC5359572

[ref12] Elias R. D.; Ma W.; Ghirlando R.; Schwieters C. D.; Reddy V. S.; Deshmukh L. Proline-rich domain of human ALIX contains multiple TSG101-UEV interaction sites and forms phosphorylation-mediated reversible amyloids. Proc. Natl. Acad. Sci. U.S.A. 2020, 117 (39), 24274–24284. 10.1073/pnas.2010635117. 32917811 PMC7533887

[ref13] Sette P.; Jadwin J. A.; Dussupt V.; Bello N. F.; Bouamr F. The ESCRT-associated protein ALIX recruits the ubiquitin ligase NEDD4–1 to facilitate HIV-1 release through the LYPXnL L domain motif. J. Virol. 2010, 84 (16), 8181–92. 10.1128/JVI.00634-10. 20519395 PMC2916511

[ref14] Korbei B. Ubiquitination of the ubiquitin-binding machinery: how early ESCRT components are controlled. Essays Biochem. 2022, 66 (2), 169–177. 10.1042/EBC20210042. 35352804 PMC9400068

[ref15] Kim T.; Chokkalla A. K.; Vemuganti R. Deletion of ubiquitin ligase NEDD4L exacerbates ischemic brain damage. J. Cereb. Blood Flow Metab. 2021, 41 (5), 1058–1066. 10.1177/0271678X20943804. 32703111 PMC8054722

[ref16] Votteler J.; Sundquist W. I. Virus budding and the ESCRT pathway. Cell Host Microbe 2013, 14 (3), 232–41. 10.1016/j.chom.2013.08.012. 24034610 PMC3819203

[ref17] Dores M. R.; Lin H.; Grimsey N. J.; Mendez F.; Trejo J. The α-arrestin ARRDC3 mediates ALIX ubiquitination and G protein–coupled receptor lysosomal sorting. Mol. Biol. Cell 2015, 26 (25), 4660–4673. 10.1091/mbc.E15-05-0284. 26490116 PMC4678022

[ref18] Hejjaoui M.; Haj-Yahya M.; Kumar K. S. A.; Brik A.; Lashuel H. A. Towards elucidation of the role of ubiquitination in the pathogenesis of Parkinson’s disease with semisynthetic ubiquitinated α-synuclein. Angew. Chem., Int. Ed. Engl. 2011, 50 (2), 405–409. 10.1002/anie.201005546. 21154793

[ref19] Haj-Yahya M.; Fauvet B.; Herman-Bachinsky Y.; Hejjaoui M.; Bavikar S. N.; Karthikeyan S. V.; Ciechanover A.; Lashuel H. A.; Brik A. Synthetic polyubiquitinated α-Synuclein reveals important insights into the roles of the ubiquitin chain in regulating its pathophysiology. Proc. Natl. Acad. Sci. U.S.A. 2013, 110 (44), 17726–17731. 10.1073/pnas.1315654110. 24043770 PMC3816408

[ref20] Meier F.; Abeywardana T.; Dhall A.; Marotta N. P.; Varkey J.; Langen R.; Chatterjee C.; Pratt M. R. Semisynthetic, site-specific ubiquitin modification of α-synuclein reveals differential effects on aggregation. J. Am. Chem. Soc. 2012, 134 (12), 5468–5471. 10.1021/ja300094r. 22404520 PMC3315850

[ref21] Elias R. D.; Ramaraju B.; Deshmukh L. Mechanistic roles of tyrosine phosphorylation in reversible amyloids, autoinhibition, and endosomal membrane association of ALIX. J. Biol. Chem. 2021, 297 (5), 101328 10.1016/j.jbc.2021.101328. 34688656 PMC8577116

[ref22] Schmidt T. G. M.; Batz L.; Bonet L.; Carl U.; Holzapfel G.; Kiem K.; Matulewicz K.; Niermeier D.; Schuchardt I.; Stanar K. Development of the Twin-Strep-tag and its application for purification of recombinant proteins from cell culture supernatants. Protein Expr. Purif. 2013, 92 (1), 54–61. 10.1016/j.pep.2013.08.021. 24012791

[ref23] Erlendsson S.; Teilum K. Binding revisited—avidity in cellular function and signaling. Front. Mol. Biosci. 2021, 10.3389/fmolb.2020.615565. PMC7841115 33521057

[ref24] Li Y.; Evers J.; Luo A.; Erber L.; Postler Z.; Chen Y. A quantitative chemical proteomics approach for site-specific stoichiometry analysis of ubiquitination. Angew. Chem., Int. Ed. Engl. 2019, 58 (2), 537–541. 10.1002/anie.201810569. 30444082

[ref25] Tofaris G. K.; Kim H. T.; Hourez R.; Jung J. W.; Kim K. P.; Goldberg A. L. Ubiquitin ligase NEDD4 promotes α-synuclein degradation by the endosomal-lysosomal pathway. Proc. Natl. Acad. Sci. U.S.A. 2011, 108 (41), 17004–9. 10.1073/pnas.1109356108. 21953697 PMC3193191

[ref26] Rodriguez J. A.; Ivanova M. I.; Sawaya M. R.; Cascio D.; Reyes F. E.; Shi D.; Sangwan S.; Guenther E. L.; Johnson L. M.; Zhang M.; Jiang L.; Arbing M. A.; Nannenga B. L.; Hattne J.; Whitelegge J.; Brewster A. S.; Messerschmidt M.; Boutet S.; Sauter N. K.; Gonen T.; Eisenberg D. S. Structure of the toxic core of α-synuclein from invisible crystals. Nature 2015, 525 (7570), 486–90. 10.1038/nature15368. 26352473 PMC4791177

[ref27] Guerrero-Ferreira R.; Taylor N. M. I.; Mona D.; Ringler P.; Lauer M. E.; Riek R.; Britschgi M.; Stahlberg H. Cryo-EM structure of α-synuclein fibrils. eLife 2018, 7, e36402 10.7554/eLife.36402. 29969391 PMC6092118

[ref28] Keren-Kaplan T.; Attali I.; Estrin M.; Kuo L. S.; Farkash E.; Jerabek-Willemsen M.; Blutraich N.; Artzi S.; Peri A.; Freed E. O.; Wolfson H. J.; Prag G. Structure-based in silico identification of ubiquitin-binding domains provides insights into the ALIX-V:ubiquitin complex and retrovirus budding. EMBO J. 2013, 32 (4), 538–551. 10.1038/emboj.2013.4. 23361315 PMC3579145

[ref29] Geiss-Friedlander R.; Melchior F. Concepts in sumoylation: a decade on. Nat. Rev. Mol. Cell Biol. 2007, 8 (12), 947–956. 10.1038/nrm2293. 18000527

