# Supplementary material for: Avidity-Based Method for the Efficient Generation of Monoubiquitinated Recombinant Proteins
Source: J Am Chem Soc. 2023 Apr 3;145(14):7748–52. doi: 10.1021/jacs.3c01943 (PMC10103170; doi:10.1021/jacs.3c01943)
Supplement: Supplementary file 1 — ja3c01943_si_001.pdf [file ja3c01943_si_001.pdf]

Avidity-based method for the efficient generation of monoubiquitinated recombinant proteins

Spencer L. Nelson[1], Yunan Li[2], Yue Chen[2], and Lalit Deshmukh[1,3]

[1]Department of Chemistry and Biochemistry, University of California San Diego, La Jolla, CA 92093, USA, [2]Department of Biochemistry, Molecular Biology, and Biophysics, University of Minnesota at Twin Cities, Minneapolis, MN 55455, USA.

[3]To whom correspondence should be addressed. Email: ldeshmukh@ucsd.edu

This file includes:

Supplementary Figures S1 to S12

Supplementary Tables S1 to S4

Supplementary References (1 to 24)

Materials and methods.

Materials.

Polyethylene glycol-4000 (PEG-4000) and PEG-8000 were purchased from Sigma-Aldrich (catalog no. 81240 and 81268, respectively). Fluorescent dyes, ATTO-488 N-hydroxysuccinimide (NHS) ester and Cy3 maleimide, were purchased from ATTO-TEC GmbH (catalog no. AD 488-31) and Cytiva (catalog no. PA13131), respectively. Both dyes were dissolved in dimethylformamide before use. Adenosine 5'-triphosphate (ATP) was purchased from Sigma-Aldrich (catalog no. A2383). α-synuclein and ALIX monoclonal primary antibodies for western blotting were purchased from Thermo Fisher Scientific (catalog no. AHB0261 and MA1-83977, respectively). The secondary antibody, IRDye 800CW, was purchased from LI-COR Biosciences (catalog no. 925-32210). Gels for sodium dodecyl sulfate polyacrylamide gel electrophoresis (SDS-PAGE) were purchased from Thermo Fisher Scientific (4–12% Bis-Tris and 3–8% Tris-Acetate gels, catalog no. NW04122BOX and EA0378BOX, respectively). Reagents for nuclear magnetic resonance (NMR) isotopic enrichment were obtained from Cambridge isotope laboratories and Sigma-Aldrich. Ingredients for NMR alignment medium, namely PEG monododecyl ether and 1-hexanol, were obtained from Sigma-Aldrich (catalog no. 76437 and H13303, respectively).

Methods.

Recombinant protein expression and purification.

Constructs used in current study, namely ubiquitinating enzymes, substrates, and modified twin-strep tagged ubiquitin, were custom synthesized from Azenta Life Sciences; see Fig. S1A for the design and Table S1 for subcloning of each construct. Constructs of tobacco etch virus (TEV) protease and wild-type ubiquitin, $Ub_{1–76}$, were generous gifts from David S. Waugh (NCI) and G. Marius Clore (NIDDK), respectively. TEV protease, $Ub_{1–76}$, and $\alpha Syn^{His}_{1–140}$ were expressed at 37 °C. All remaining constructs were expressed at 16 °C. Cells were grown at 37 °C in 1 L Luria-

Bertani (LB; MP Biomedicals, catalog no. 3002-036) medium or Terrific Broth (TB; Thermo Fisher Scientific, catalog no. BP9728-500) at natural isotopic abundance or minimal M9 medium[1-2] for isotopic labeling. Cells were induced with 1 mM isopropyl β-d-1-thiogalactopyranoside (IPTG) and 0.2% (w/v) arabinose at an absorbance of 0.8 at 600 nm; note that arabinose was used in the case of BL21-AI competent cells.

The purification scheme of TEV protease has been described previously.[3] The enzymes UbE1, UbE2D3, UbE3-NEDD4L, and UbE3-WWP2 were purified using a combination of affinity and size-exclusion chromatography (ÄKTA Pure and Start protein purification systems, Cytiva). Briefly, cells were resuspended in a lysis buffer containing 50 mM Tris, pH 8.0, and 250 mM NaCl. Cells were lysed using EmulsiFlex-C3 (Avestin), and cleared by centrifugation (48,380g, 30 min). The cell lysates were loaded onto HisTrap columns (Cytiva). The bound enzymes were washed with 10 column volumes of the lysis buffer, followed by 10 column volumes of 30 mM imidazole, and eluted in the same buffer containing 500 mM imidazole. The eluted enzymes were concentrated (Amicon ultra-15 centrifugal filter units, 10/30 kDa cutoff; EMD Millipore) and loaded onto HiLoad 26/600 Superdex 200 or HiLoad 26/600 Superdex 75 prep-grade columns (Cytiva) pre-equilibrated with 50 mM Tris, pH 8.0, 150 mM NaCl, 1 mM ethylenediaminetetraacetic acid (EDTA), and 1 mM dithiothreitol (DTT). The eluted enzymes were mixed with recombinant TEV protease (molar ratio 50:1) to hydrolyze polyhistidine affinity tags (total incubation time: ~ 20 h at room temperature). The subsequent reaction mixtures were loaded onto HisTrap columns, and the relevant flow-through fractions of TEV-cleaved enzymes were pooled, concentrated, and loaded onto HiLoad 26/600 Superdex 200 or HiLoad 26/600 Superdex 75 prep-grade columns pre-equilibrated with 50 mM Tris, pH 8.0, 150 mM NaCl, 1 mM DTT, and 1 mM EDTA. Relevant UbE1/UbE2D3 fractions were concentrated (~5 mg/mL) and kept at -20 °C, whereas UbE3-NEDD4L/WWP2 fractions were pooled and kept at 4 °C.

For $\alpha\mathrm{Syn}^{\mathrm{His}}_{1-140}$, cells were resuspended in a lysis buffer containing 50 mM Tris, pH 8.0. Cells were lysed and cleared by centrifugation. The cell lysate was loaded onto a HisTrap column. The bound

protein was washed with 10 column volumes of the lysis buffer and eluted in the same buffer containing 500 mM imidazole. The eluted protein was loaded onto a Mono Q 10/100 GL anion-exchange chromatography column (Cytiva) with a 0–1 M NaCl gradient in a buffer containing 50 mM Tris, pH 8.0, and 1 mM EDTA. The eluted protein was further purified using high-performance liquid chromatography (HPLC; Jupiter 10 μm C18 300 Å column, Phenomenex; catalog no. 00G-4055-N0) with a 25–70% acetonitrile gradient comprising 0.1% trifluoroacetic acid (TFA; Sigma-Aldrich). The eluted fractions of $\alpha\mathrm{Syn}_{1-140}^{\mathrm{His}}$ were freeze-dried (Labconco -84 °C benchtop freeze dryer) and stored at -80 °C.

Cells expressing $\mathrm{ALIX}_{1-868*}^{\mathrm{His}}$, resuspended in a lysis buffer containing 50 mM Tris, pH 8.0, and 250 mM NaCl, were lysed and cleared by centrifugation. The cell lysate was loaded onto a HisTrap column. The bound protein was washed with 10 column volumes of the lysis buffer, 10 column volumes of 30 mM imidazole, and eluted in the same buffer containing 500 mM imidazole. The eluted protein was concentrated (Amicon ultra-15 centrifugal filter units, 30-kDa cutoff) and loaded onto a HiLoad 26/600 Superdex 200 pg column pre-equilibrated with 50 mM Tris, pH 8.0, 150 mM NaCl, 1 mM DTT, and 1 mM EDTA. Relevant fractions of $\mathrm{ALIX}_{1-868*}^{\mathrm{His}}$ were concentrated (~20 mg/mL) and stored at -20 °C.

For $\mathrm{ALIX}_{348\text{-}702}$, a similar protocol as to that of $\mathrm{ALIX}_{1-868*}^{\mathrm{His}}$ was used. Briefly, the protein was purified using a combination of nickel affinity and size-exclusion chromatography. The eluted protein from the sizing (HiLoad 26/600 Superdex 75 prep-grade) column was mixed with TEV protease to hydrolyze the B1 domain of protein G (GB1)-polyhistidine fusion tag.[4] The subsequent reaction mixture was loaded onto a HisTrap column, and the flow-through fractions of TEV-cleaved protein were pooled, concentrated, and loaded onto a HiLoad 26/600 Superdex 75 prep-grade column pre-equilibrated with 50 mM Tris, pH 8.0, 250 mM NaCl, and 1 mM DTT. Relevant fractions were pooled and kept at 4 °C.

Cells of $\mathrm{Ub}_{1-76}^{\mathrm{Strep(GS)_2}}$, resuspended in a lysis buffer containing 50 mM Tris, pH 8.0, 150 mM NaCl, and 1 mM EDTA, were lysed and cleared by centrifugation. The cell lysate was loaded onto an

XK 16/20 chromatography column (Cytiva) prepacked with Strep-Tactin XT Sepharose resin (Cytiva). The bound protein was washed with 10 column volumes of the lysis buffer and eluted in the same buffer containing 50 mM biotin. The eluted protein was further purified using reverse-phase HPLC (Jupiter 10 µm C18 300 Å column, Phenomenex) with a 25–49% acetonitrile gradient comprising 0.1% TFA. The eluted fractions of $Ub_{1-76}^{Strep(GS)_2}$ were freeze-dried and stored at -20 °C. To hydrolyze modified twin-strep tag, lyophilized $Ub_{1-76}^{Strep(GS)_2}$ was reconstituted in ~100 µL buffer comprising 6 M guanidinium hydrochloride and 50 mM Tris, pH 8.0. Protein was exchanged into a buffer containing 50 mM Tris pH 8.0 and 1 mM DTT and subsequently mixed with TEV protease. The hydrolyzed product was purified using a combination of affinity chromatography and reverse-phase HPLC. The eluted fractions of $Ub_{1-76}^{(GS)_2}$ were freeze-dried and stored at -80 °C.

For wild-type ubiquitin, Ub_{1-76}, the cell lysate comprising 50 mM Tris (pH 7.5) was cleared by centrifugation and subsequently mixed with perchloric acid. The resultant mixture (pH 4) was further cleared by centrifugation and loaded on a HiLoad 16/10 SP Sepharose HP column (Cytiva) pre-equilibrated in a buffer containing 50 mM sodium acetate, pH 4.5. The bound protein was eluted in the same buffer comprising a 0–1 M NaCl gradient. Relevant fractions were pooled and further purified using reverse-phase HPLC (Jupiter 10 µm C18 300 Å column, Phenomenex) with a 5–75% acetonitrile gradient comprising 0.1% TFA. The eluted protein was collected, freeze-dried, and stored at -20 °C until use.

All protein constructs and monoubiquitinated αSyn_{1-140}^{His} and $ALIX_{1-868*}^{His}$ were verified by mass spectrometry (MS) using our previously described protocol.[1-3]

In vitro ubiquitination and purification of monoubiquitinated products.

Ubiquitinating enzymes (UbE1, UbE2D3, and UbE3-NEDD4L/WWP2), $Ub_{1-76}^{Strep(GS)_2}$, and substrate (αSyn_{1-140}^{His} / $ALIX_{1-868*}^{His}$) were mixed in a reaction buffer comprising 50 mM Tris, pH 7.5, 1 mM DTT, and 1 mM $MgCl_2$, Table S2. The reaction mixture was dialyzed against the same buffer with the addition of 1.5 mM ATP (total incubation time: 5 h at 30 °C). The resultant reaction was cleared by centrifugation and loaded onto a HisTrap column pre-equilibrated with a buffer containing 50

mM Tris, pH 8.0, and 250 mM NaCl. The bound components (i.e., the substrate and its ubiquitinated products) were eluted with 500 mM imidazole. The eluted proteins were loaded onto a XK 16/20 chromatography column prepacked with Strep-Tactin XT Sepharose resin (Cytiva) pre-equilibrated with buffer containing 50 mM Tris, pH 8.0, 150 mM NaCl, and 1 mM EDTA. The bound protein was eluted in the same buffer comprising a 0–50 mM biotin gradient. Relevant monoubiquitinated protein fractions were pooled and stored at -20 °C. The reaction progress and the subsequent purification of monoubiquitinated moieties were assessed using SDS-PAGE and western blotting using our published protocol.[1-2] Blots were visualized using the Odyssey XF imaging system (LI-COR Biosciences).

For monoubiquitinated $\alpha\mathrm{Syn}_{1-140}^{\mathrm{His}}$, the modified twin-strep tag of $\mathrm{Ub}_{1-76}^{\mathrm{Strep(GS)_2}}$ was hydrolyzed using TEV protease. The resultant mixture was purified using reverse-phase HPLC (Jupiter 10 µm C18 300 Å column, Phenomenex) with a 35–45% acetonitrile gradient comprising 0.1% TFA. The eluted monoubiquitinated protein, $\mathrm{Ub}_{1-76}^{\mathrm{(GS)_2}}$-$\alpha\mathrm{Syn}_{1-140}^{\mathrm{His}}$, was collected, freeze-dried, and stored at -20 °C. These samples were used to determine the impact of monoubiquitination on the aggregation properties of α-synuclein. A similar procedure was not carried out for monoubiquitinated ALIX, $\mathrm{Ub}_{1-76}^{\mathrm{Strep(GS)_2}}$-$\mathrm{ALIX}_{1-868*}^{\mathrm{His}}$, since the tag represented a minimal portion of the protein (3.45 kDa for the tag vs. 109.6 kDa for the monoubiquitinated ALIX).

Quantitative chemical proteomics.

Quantitative chemical proteomics was performed as previously described,[5] and carried out on two independent in vitro ubiquitination reactions of $\alpha\mathrm{Syn}_{1-140}^{\mathrm{His}}$ and $\mathrm{ALIX}_{1-868*}^{\mathrm{His}}$. For each reaction, quantitative chemical proteomics was carried out in triplicate by dividing the product into three. Briefly, ~5 µg of monoubiquitinated $\alpha\mathrm{Syn}_{1-140}^{\mathrm{His}}$ and $\mathrm{ALIX}_{1-868*}^{\mathrm{His}}$ were resuspended in 10 µL of 9 M urea in phosphate buffered saline (PBS). A labeling solution (100 mg/mL in 50% acetonitrile + 50% water) comprising amine-reactive chemical tag, acetyl glycylglycine-NHS (Ac-GG-NHS), was added to the samples with 1 µL each time and vortexing for 45 min. The labeling was repeated for a total of three times while maintaining a pH of 8–8.5. The reaction was quenched with the

addition of 5% hydroxylamine (pH 6.0, 1.5 M) for 15 min. Samples were diluted by 1x PBS for six folds and digested by trypsin (Promega Corporation) at 37 °C overnight, followed by a second digestion with endoproteinase Glu-C (Sigma-Aldrich) for overnight at an enzyme-to-substrate ratio of 1:10 (w/w). Digested peptides were desalted with home-made C18 StageTip and dried in a SpeedVac vacuum concentrator (Thermo Fisher Scientific). Peptides were resuspended in 10 µl of 9 M urea in PBS. A labeling solution comprising heavy acetyl($^{13}CD_3$-^{13}CO)-NHS (100 mg/mL in 100% acetonitrile) was added to the peptide samples for the second labeling with 1 µL each time and vortexing for 45 min. The labeling was repeated for a total of three times and quenched with 5% hydroxylamine (pH 6.0, 1.5 M) for 15 min. The peptides were then desalted with StageTip and subjected to liquid chromatography (LC)–MS analysis (see below).

Peptides were dissolved in HPLC buffer A (0.1% formic acid in water) and injected into Dionex Ultimate 3000 RSLCnano-UPLC system (Thermo Fisher Scientific). Peptides were separated with a gradient of 5–90% HPLC buffer B for 53.5 min (0.1% formic acid in acetonitrile) on a home-packed C18 (Luna 5 µm, 100Å pores; Phenomenex) capillary reverse-phase HPLC column (20 cm in length and 75 µm in internal diameter) with an integrated emission tip (New Objective, Inc.) and electrosprayed to the Orbitrap Fusion mass spectrometer (Thermo Fisher Scientific). The MS instrument was operated in a data-dependent mode with one full MS scan in Orbitrap at a resolution of 60,000 (200 m/z) followed by data-dependent MS/MS in linear ion trap with a stepped high-energy collision dissociation (HCD) at 35% and an isolation window of 1.2 m/z. LC–MS data were analyzed by MaxQuant software (version 1.5.3.12)[6] and searched against the protein sequence database of the target monoubiquitinated proteins ($\alpha\mathrm{Syn}^{\mathrm{His}}_{1\text{–}140}$ and $\mathrm{ALIX}^{\mathrm{His}}_{1\text{–}868*}$) concatenated with common contamination proteins. Trypsin and Glu-C were specified as proteolytic enzymes, acetylation on protein N-terminus, oxidation on methionine, heavy acetyl ($^{13}CD_3$-^{13}CO) modification on peptide N-terminus, Ac-GG modification on protein N-terminus, Ac-GG and heavy Ac($^{13}CD_3$-^{13}CO)-GG modification on lysine were specified as variable modifications and cysteine carbamidomethylation were specified as a fixed modification. False discovery rate of 1%

with the target-decoy strategy was applied for all peptide and protein identifications. Peptides identified with Ac-GG labeling were manually analyzed using Xcalibur (version 4.1; Thermo Fisher Scientific) to quantify the heavy and light ubiquitinated peptide forms and ubiquitination stoichiometry as previously described.[5] Additionally, similar measurements and analyses were performed on NEDD4L-mediated multi-mono/polyubiquitinated $\alpha\text{Syn}^{\text{His}}_{1\text{–}140}$ samples to determine the subsequent changes in ubiquitination frequencies.

Fluorophore labeling.

$\alpha\text{Syn}^{\text{His}}_{1\text{–}140}$ was mixed with a 4-molar equivalent of ATTO-488 NHS ester in 20 mM sodium carbonate buffer (pH 8.3); total incubation time: ~1 h at room temperature. Unreacted dye was removed using a PD MidiTrap G-25 desalting column (Cytiva), and the buffer was exchanged to 25 mM Tris, pH 7.4, 50 mM NaCl, and 1 mM EDTA.

$\text{ALIX}^{\text{His}}_{1\text{–}868*}$ was mixed with 10-molar equivalents of Cy3 maleimide in 25 mM HEPES, pH 7.4, 150 mM NaCl, 1 mM tris(2-carboxyethyl)phosphine [TCEP], and 1 mM EDTA; total incubation time: ~2 h at room temperature, followed by 4 °C overnight. Unreacted dye was removed using a PD MidiTrap G-25 desalting column.

Phase separation.

$\alpha\text{Syn}^{\text{His}}_{1\text{–}140}$ and its monoubiquitinated counterpart were dialyzed in 25 mM Tris, pH 7.4, 50 mM NaCl, and 1 mM EDTA. Phase separation was initiated by the addition of 10% (w/v) PEG-8000. In all samples, the protein concentration was maintained at 200 μM. For $\text{ALIX}^{\text{His}}_{1\text{–}868*}$, unmodified and monoubiquitinated proteins were dialyzed in 25 mM HEPES, pH 7, 150 mM NaCl, 1 mM DTT, and 1 mM EDTA. Phase separation was initiated by the addition of 5% (w/v) PEG-4000. In all samples, the protein concentration was maintained at 50 μM.

NMR.

Samples of ^{15}N-labeled or ^{15}N/^{13}C-labeled ubiquitin variants, namely $\text{Ub}_{1\text{–}76}$, $\text{Ub}^{\text{Strep(GS)}_2}_{1\text{–}76}$, and $\text{Ub}^{\text{(GS)}_2}_{1\text{–}76}$, were prepared in a buffer comprising 20 mM sodium phosphate, pH 6.5, and 1 mM EDTA.

A similar buffer composition with the addition of 1 mM TCEP was used for NMR titration experiments (see below). An aligned sample of ^{15}N-labeled $Ub_{1-76}^{Strep(GS)_2}$ was prepared using 5% PEG-hexanol.[7] All NMR samples contained 7% (v/v) deuterium oxide.

NMR experiments were carried out at 27 °C on Bruker 600 and 800 MHz spectrometers equipped with z-gradient triple resonance cryoprobes. Spectra were processed using NMRPipe[8] and analyzed using the CCPN software suite.[9] Sequential ^{1}H, ^{15}N, and ^{13}C backbone resonance assignments of ubiquitin variants were carried out using transverse relaxation optimized spectroscopy (TROSY)-based three-dimensional (3D) triple resonance experiments.[10] NMR chemical shift perturbation experiments were performed using 0.1 mM ^{15}N-labeled Ub_{1-76} and unlabeled $ALIX_{348-702}$ / $ALIX_{1-868*}^{His}$ (0.1 mM each). Perturbations were calculated as follows: $\Delta_{H/N} = \{(\Delta\delta_{HN})^2 + (0.154 \times \Delta\delta_N)^2\}^{1/2}$, where $\Delta\delta_{HN}$ and $\Delta\delta_N$ are the $^{1}H_N$ and ^{15}N chemical shift differences in ppm, respectively, between free and bound states. $^{1}D_{NH}$ residual dipolar couplings (RDCs), given by the difference in $^{1}J_{NH}$ coupling constants in aligned and isotropic media, were measured using the TROSY-based ARTSY technique[11] and analyzed with Xplor-NIH.[12] To identify chemical shift perturbations upon monoubiquitination, in-vitro ubiquitination reactions were carried out using ^{15}N-labeled $Ub_{1-76}^{Strep(GS)_2}$ and unlabeled αSyn_{1-140}^{His}. The corresponding monoubiquitinated αSyn_{1-140}^{His} was analyzed using a two-dimensional (2D) TROSY-HSQC experiment and was compared against TROSY-HSQC spectrum of ^{15}N-labeled free $Ub_{1-76}^{Strep(GS)_2}$. Perturbations were calculated using the above-described formula.

Microscopy.

Glass coverslips (VWR; catalog no. 48366-172) were passivated[13] using the following protocol. Coverslips were heated and sonicated for 10 minutes in 1% Hellmanex III solution (Sigma-Aldrich, catalog no. Z805939). Coverslips were rinsed with 0.1 M NaOH and water. Cleaned coverslips were dried with nitrogen, rinsed with acetone, and dried again followed by the application of 5% (w/v) mPEG20K-Silane (Sigma-Aldrich, catalog no. JKA3100) in dimethyl

sulfoxide. Coverslips were then incubated at 90 °C for 20 min. Passivated coverslips were rinsed with water and dried with nitrogen before use.

Condensate samples for differential interference contrast (DIC) microscopy were applied to passivated coverslips (see above) and incubated for 5 min at room temperature to allow condensates to settle onto the surface before being sandwiched by another passivated coverslip. DIC microscopy was performed on a Nikon Ti2 widefield microscope equipped with a DS-Qi2 CMOS camera and a 6x/1.4NA oil DIC N2 Objective. The samples were excited by a 470/555 nm laser controlled by a Lumencor SpectraX for imaging of ATTO-488, and Cy3, respectively. Condensate samples for fluorescence recovery after photobleaching (FRAP) assays were applied to passivated coverslips with silicone gaskets (EMD-Millipore; catalog no. GBL664112) and allowed to incubate for 5 min at room temperature before sealing the gasket with an additional coverslip. FRAP experiments were performed on a Nikon point scanning confocal C2 with 2 GaAsP PMTs using a Plan Apo λ 60x/1.4 NA oil objective. Data collection consisted of two pre-photobleaching frames excited at 0.5% 488 nm laser power, followed by photobleaching with 2 iterations of 10% 488 nm laser power directed at the bleaching area for 4 s, and subsequently excited at 0.2% 488 nm laser power at 0.5 s intervals for 302 frames as post-photobleaching frames. Images were corrected for background fluorescence and intensity from the bleached region was normalized against the intensity of prebleached droplets.

Timelapse for the fusion of condensates of $\alpha\text{Syn}^{\text{His}}_{1-140}$ and its monoubiquitinated counterpart were performed at room temperature in 25 mM Tris, pH 7.4, 50 mM NaCl, 1 mM EDTA and 10% (w/v) PEG-8000, with 200 μM proteins (10% ATTO-488 labeled and 90% unlabeled). Images were collected every 60 s over the course of 142 minutes using a 488 nm laser on an EVOS M5000 (Thermo Fisher Scientific) using a Plan Apo λ 60x/1.4 NA oil objective (Olympus).

Thioflavin T (ThT) aggregation assays.

ThT aggregation assays of $\alpha\text{Syn}^{\text{His}}_{1-140}$ and its monoubiquitinated counterpart were performed in buffer containing 50 mM sodium acetate, pH 5.1, 1 mM EDTA, and 20 μM ThT (protein

concentration = 30 μM). Samples were incubated at 37 °C in sealed 96-well flat bottom plates (Corning, catalog no. 3370) containing 100 μL sample per well (n = 2). Measurements were carried out with 3 min of orbital shaking (2 mm, 280 rpm) and 2 min of rest using a microplate reader (Infinite M Plex, Tecan). ThT fluorescence was recorded as a function of time (every 5 min); excitation and emission wavelengths were 415 and 480 nm, respectively.

Monoubiquitinated $ALIX^{His}_{1-868*}$ was allowed to aggregate for a few days to create seeds.[14] Seeds were resuspended in a buffer comprising 25 mM HEPES, 150 mM NaCl, 1 mM EDTA, and 1 mM DTT before addition to the solutions of fresh monoubiquitinated $ALIX^{His}_{1-868*}$ and its unmodified counterpart (100 μM each). Seeds represented ~5% of the total protein mass. ThT fluorescence (20 μM) was recorded every 10 min with continuous shaking (the remaining parameters were same as above).

Transmission electron microscopy (TEM).

TEM samples of α-synuclein and ALIX fibrils were prepared using our published protocol.[15] TEM images were acquired using a JEM-1400Plus transmission electron microscope (JEOL) and recorded on a OneView digital camera (Gatan).

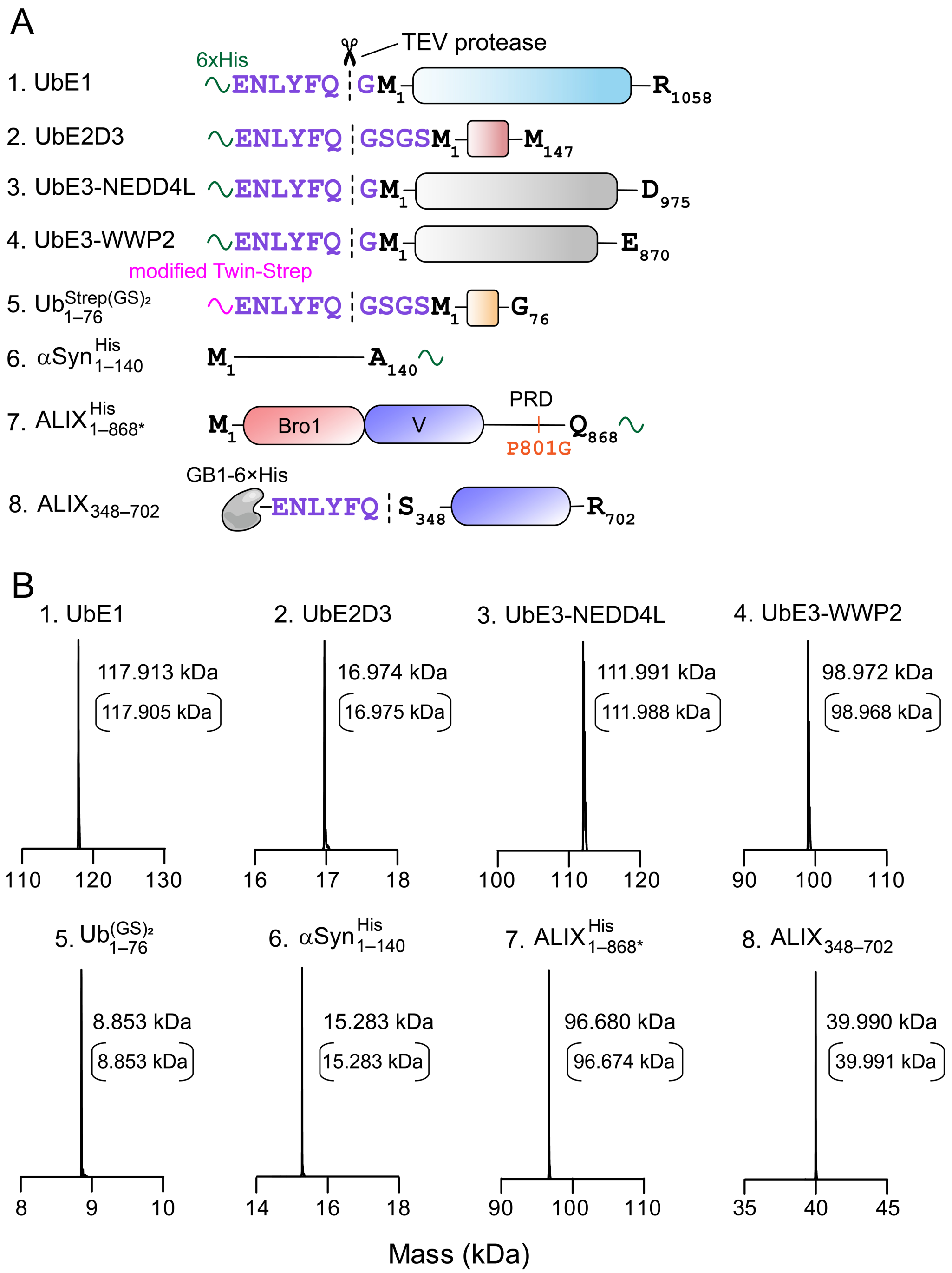

Figure S1. Recombinant constructs used in current study. (**A**) List of proteins used in current study. Each protein construct is designated by a number; see Table S1 for expression conditions, UniProt, and Addgene entries of each construct. All constructs were custom synthesized from Azenta Life Sciences. The native residues at the N- and C-termini of each construct are labeled in black. The primary sequences of TEV protease cleavage sites are labeled in purple. The locations of the TEV cleavage sites are marked by vertical dashed lines and scissors. 6xHis (green ribbon) denotes a polyhistidine affinity tag comprising six

Figure S1 (cont'd). histidine residues. For $Ub^{Strep(GS)_2}_{1-76}$ (construct 5), the primary sequence of the modified twin-strep-tag is as follows: MGSWSHPQFE**R**(GGGS)$_2$GGS**S**AWSHPQFE**R**GS. This sequence differs from Schmidt and colleagues' original twin-strep-tag sequence[16] in the following two ways: (1) the bold and underlined arginine residues are mutated from the original sequence's lysine residues to prevent spurious ubiquitination, and (2) an extra serine residue (bold and underlined) was incorporated to increase bacterial expression. Both substrates, αSyn^{His}_{1-140} and $ALIX^{His}_{1-868*}$ (constructs 6 and 7, respectively), carry non-cleavable C-terminal 6xHis affinity tags. For $ALIX^{His}_{1-868*}$, the asterisk denotes the P801G point-mutation (orange) that was required for its overexpression in E. coli (Elias et al., manuscript submitted).[17] For $ALIX_{348-702}$ (construct 8), GB1–6xHis denotes protein GB1,[4] used to enhance protein expression levels, followed by a spacer sequence, and a polyhistidine affinity tag. (**B**) Analysis of TEV-cleaved recombinant proteins using liquid chromatography–electrospray ionization–time-of-flight mass spectrometry (LC–ESI–TOFMS); the numbers in parenthesis represent the corresponding theoretical masses.

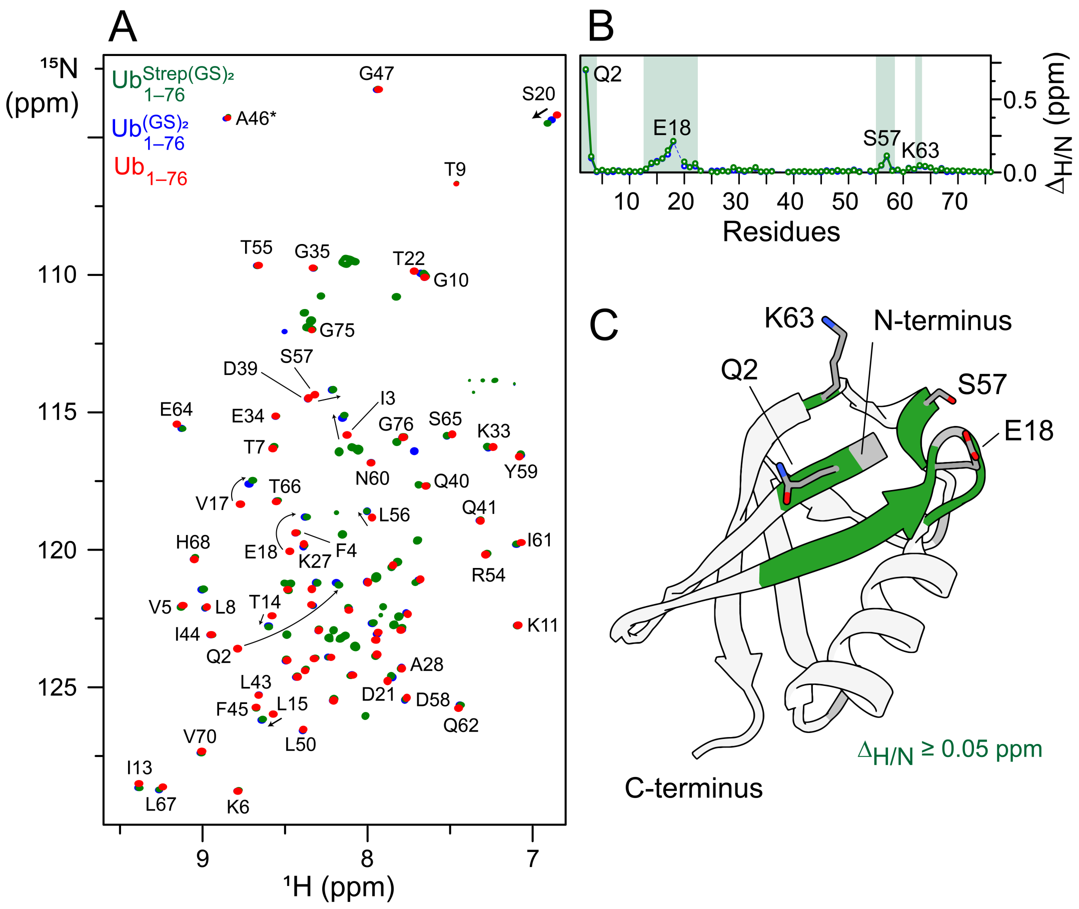

Figure S2. NMR chemical shift analysis of ubiquitin constructs used in current study. (**A**) Overlay of the expanded regions of ^{1}H–^{15}N TROSY–HSQC spectra of $Ub_{1-76}^{Strep(GS)_2}$, $Ub_{1-76}^{(GS)_2}$, and Ub_{1-76} (green, blue, and red, respectively). A few of the ^{1}H–^{15}N cross-peaks of Ub_{1-76} are labeled (folded cross-peaks of residue A46 are marked by an asterisk). The changes in chemical shifts of the ^{1}H–^{15}N cross-peaks due to the presence of the N-terminal modified twin-strep tag and/or GSGS linker remnant of the TEV cleavage site (cf. Fig. S1A) are marked by arrows. (**B**) $^{1}H_N$/^{15}N chemical shift perturbation profiles of Ub_{1-76} vs. $Ub_{1-76}^{Strep(GS)_2}$, and Ub_{1-76} vs. $Ub_{1-76}^{(GS)_2}$ (green and blue, respectively). Semi-transparent green rectangles indicate residues (2–3, 14–22, 56–57, and 63) that exhibit large chemical shift perturbations (≥0.05 ppm). A few residues are labeled. (**C**) Ribbon diagram of ubiquitin (PDB entry: 1UBQ)[18]; a few of the residues that undergo large chemical shift changes due to N-terminal modifications (i.e., modified twin-strep tag and/or GSGS linker) are shown in stick representation. Green ribbons represent residues that are most affected ($\Delta_{H/N} \geq 0.05$ ppm). Grey ribbons represent residues that could not be assigned unambiguously and the N-terminal methionine. The experimental data were recorded at 27 °C and a spectrometer ^{1}H frequency of 600 MHz.

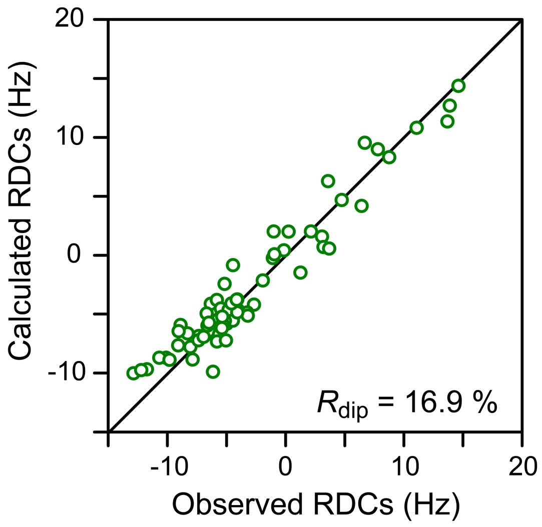

Figure S3. Backbone RDC analysis of $Ub_{1-76}^{Strep(GS)_2}$. Singular value decomposition (SVD) analysis showing agreement of the experimental backbone amide ($^1D_{NH}$) RDCs acquired in 5% PEG-hexanol with those calculated from the X-ray coordinates of ubiquitin (PDB entry: 1UBQ)[18]. Only residues in secondary structure elements are used for the SVD fit. The RDC R-factor, R_{dip}, is given by $\{<(D_{obs}-D_{calc})^2>/(2<D_{obs}{}^2>)\}^{1/2}$, where D_{obs} and D_{calc} are the observed and calculated $^1D_{NH}$ RDC values, respectively.[19] Excellent agreement between observed and calculated RDC values with R_{dip} of ~17% indicate that $Ub_{1-76}^{Strep(GS)_2}$ adopts the same fold in solution as that of wild-type ubiquitin. The experimental data were recorded at 27 °C and a spectrometer ^{1}H frequency of 800 MHz. The concentrations of proteins in aligned and isotropic samples were 0.2 mM each.

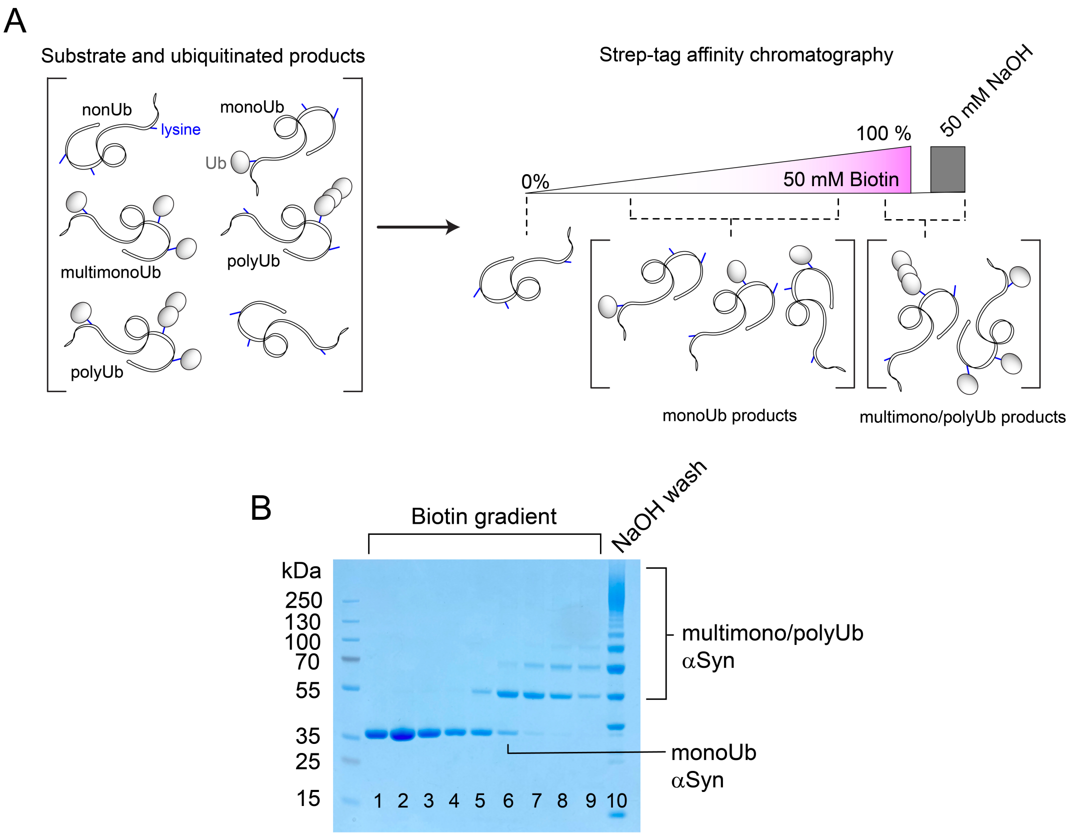

Figure S4. Exploiting the avidity effect for the selective purification of monoubiquitinated species. (**A**) Reaction components from Step-3 (see Fig. 1A, main text) comprising substrate and its ubiquitinated products were subjected to Strep-tag affinity chromatography. Components carrying a single $Ub_{1-76}^{Strep(GS)_2}$ molecule comprising a modified twin-strep tag, i.e., monoubiquitinated products, bound efficiently to the tetrameric strep-tactin, a derivative of streptavidin. These monoubiquitinated species could, however, be readily displaced from the resin-coupled strep-tactin by biotin. In contrast, components carrying two or more $Ub_{1-76}^{Strep(GS)_2}$ molecules, namely multi-mono- and poly-ubiquitinated products, remained tightly bound to the resin due to the avidity effect and could only be displaced using 50 mM biotin or 50 mM sodium hydroxide (NaOH). The latter was used to regenerate the resin for the next use.[16, 20] This is because the binding affinity between streptavidin and its derivatives to biotin and related ligands is severely diminished in the presence of 50 mM NaOH (pH ~12.7) and can be recovered to normal levels by re-equilibration with neutral buffer.[21] Note that the avidity effect, which we define as the increased strength of binding due to multiple interactions, likely originates from the simultaneous binding of multiple twin-strep tags of multi-mono- and poly-ubiquitinated products to the tetrameric strep-tactin. (**B**) A representative SDS-PAGE gel showing the effectiveness of this purification scheme and the corresponding NaOH wash (lane 10); a prep of ubiquitinated αSyn_{1-140}^{His} was used to generate this gel.

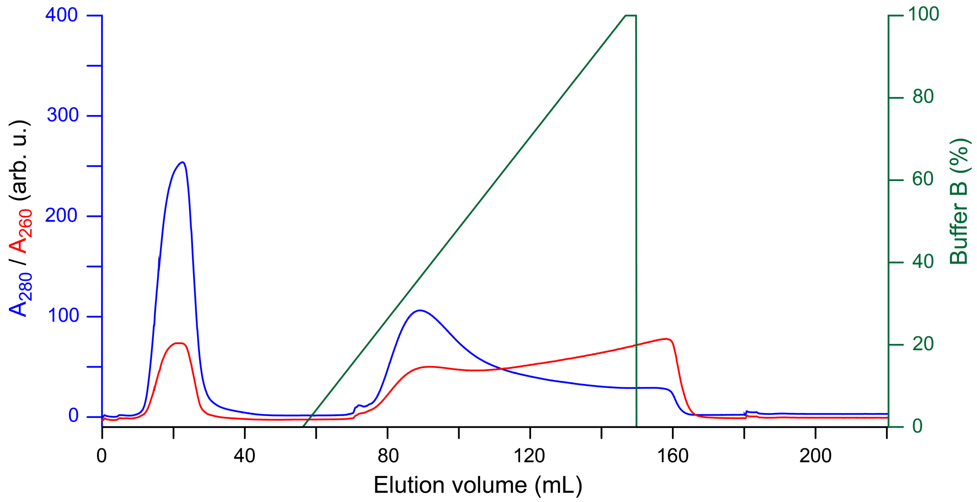

Figure S5. Strep-tag affinity chromatography for a selective purification of monoubiquitinated moieties. A prep of ubiquitinated $ALIX^{His}_{1–868*}$ (with NEDD4L) was used to generate this chromatogram. The composition of the running buffer was as follows: 50 mM Tris, pH 8.0, 150 mM NaCl, and 1 mM EDTA. The bound monoubiquitinated $ALIX^{His}_{1–868*}$ was eluted in the same buffer comprising a 0–50 mM biotin gradient (Buffer B).

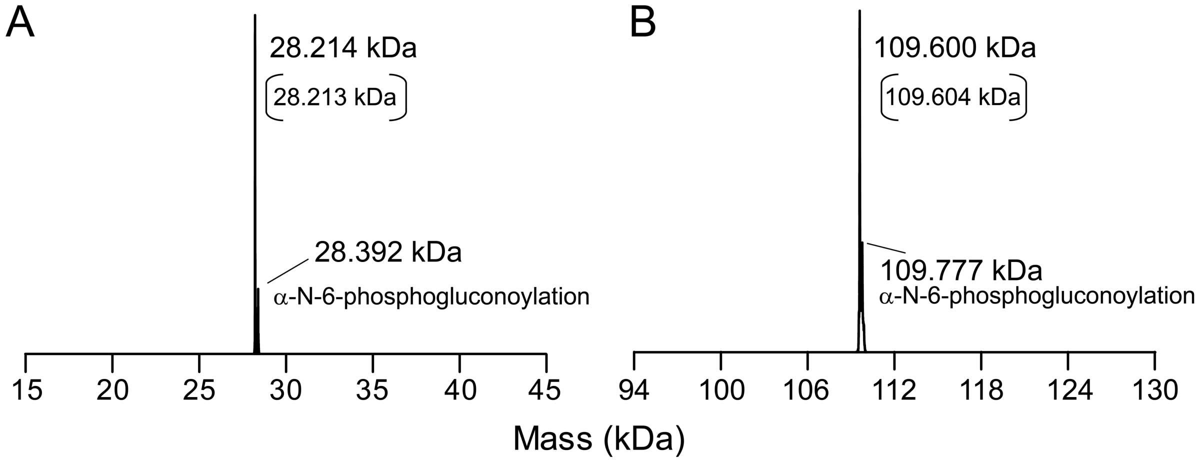

Figure S6. Mass-spectrometry analysis of monoubiquitinated α-synuclein and ALIX. LC–ESI–TOFMS analyses of monoubiquitinated (**A**) αSyn^{His}_{1-140} and (**B**) $ALIX^{His}_{1-868*}$. NEDD4L and WWP2 were used for monoubiquitination of α-synuclein and ALIX, respectively. Also see Fig. 1B–C (main text) for the corresponding western blots and SDS-PAGE gel analyses. The numbers in parenthesis represent theoretical masses calculated using the formula: mass of intact $Ub^{Strep(GS)_2}_{1-76}$ (12.948 kDa; note that Fig. S1B reports the mass of TEV-cleaved $Ub^{(GS)_2}_{1-76}$) + mass of the substrate (15.283 kDa for αSyn^{His}_{1-140} / 96.674 kDa for $ALIX^{His}_{1-868*}$) – 0.018 kDa (representing the loss of water molecule owing to the formation of the isopeptide bond). Minor peaks (representing approximately 178 Da addition) are most likely caused by α-N-6-phosphogluconoylation[22] of the modified twin-strep tag of $Ub^{Strep(GS)_2}_{1-76}$.

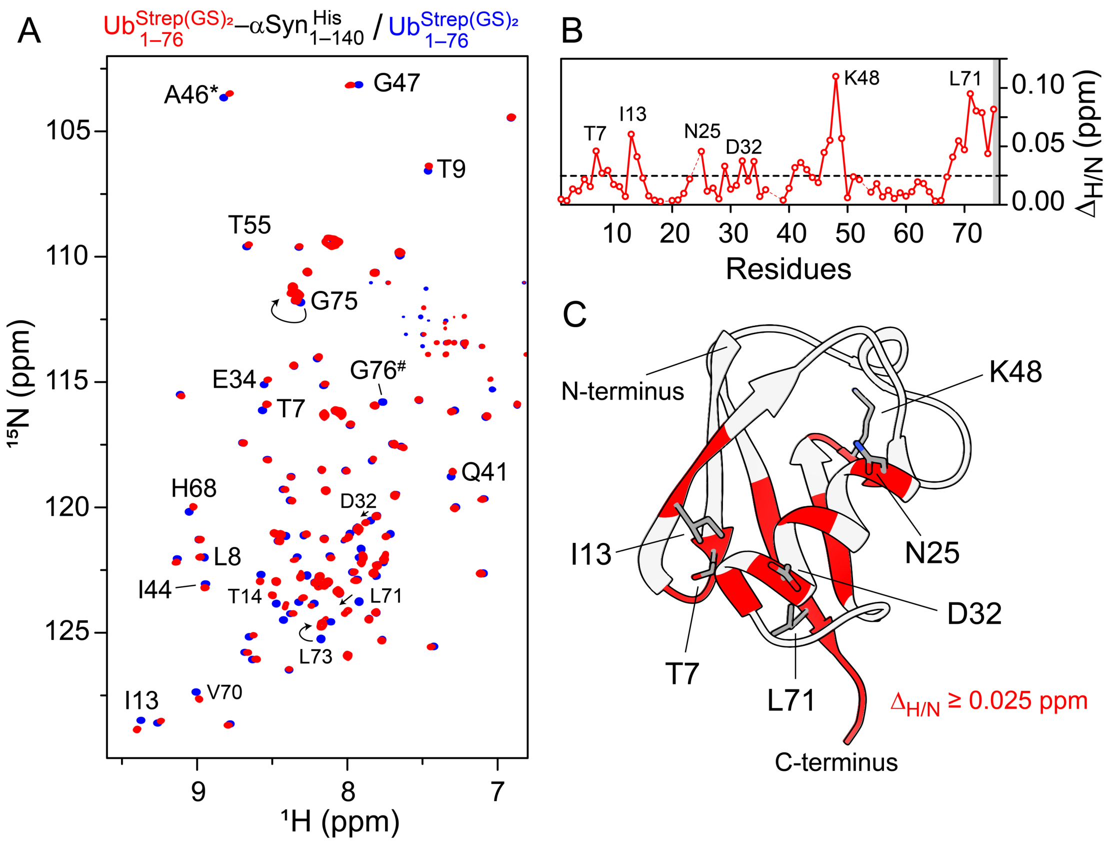

Figure S7. Solution NMR analysis of monoubiquitinated α-synuclein. (**A**) Overlay of the expanded regions of ^{1}H–^{15}N TROSY–HSQC spectra of $\mathrm{Ub}_{1–76}^{\mathrm{Strep(GS)_2}}$–$\alpha\mathrm{Syn}_{1–140}^{\mathrm{His}}$ and $\mathrm{Ub}_{1–76}^{\mathrm{Strep(GS)_2}}$ (red and blue, respectively). The corresponding in vitro ubiquitination reaction was carried out using NEDD4L and ^{15}N-labeled (NMR-visible) $\mathrm{Ub}_{1–76}^{\mathrm{Strep(GS)_2}}$ and unlabeled (NMR-invisible) $\alpha\mathrm{Syn}_{1–140}^{\mathrm{His}}$. A few of the ^{1}H–^{15}N cross-peaks that show large chemical shift perturbations are labeled (the numbering is based on $\mathrm{Ub}_{1–76}$). The folded cross-peaks of A46 are marked by an asterisk. Residue G76 exhibited resonance line broadening upon monoubiquitination (marked by #). (**B**) $^{1}\mathrm{H_N}$/^{15}N chemical shift perturbation profiles of $\mathrm{Ub}_{1–76}^{\mathrm{Strep(GS)_2}}$–$\alpha\mathrm{Syn}_{1–140}^{\mathrm{His}}$ vs. $\mathrm{Ub}_{1–76}^{\mathrm{Strep(GS)_2}}$. Semi-transparent gray rectangle marks the location and disappearance of G76 cross-peak upon monoubiquitination. A few residues that show large perturbations are labeled. (**C**) Ribbon diagram of ubiquitin (PDB entry: 1UBQ)[18]. Red ribbons represent residues that show large perturbations in **B** ($\Delta_{\mathrm{H/N}} \geq 0.025$ ppm). A few affected residues are shown in stick representation. All spectra were recorded at a spectrometer ^{1}H frequency of 800 MHz. The experimental conditions were as follows: 20 mM sodium phosphate, pH 6.5, 1 mM EDTA, and 1 mM TCEP at 27 °C. The concentrations of proteins were 0.1 mM each. Note that a similar reaction was carried out using unlabeled (NMR-invisible) $\mathrm{Ub}_{1–76}^{\mathrm{Strep(GS)_2}}$ and ^{15}N-labeled (NMR-visible) $\alpha\mathrm{Syn}_{1–140}^{\mathrm{His}}$. No discernible differences in chemical shifts between the cross-peaks of modified and unmodified α-synuclein samples were observed. This is because the observed chemical shifts

Figure S7 (cont'd). are population weighted averages, and heterogeneous monoubiquitination of α-synuclein (cf. Fig. 2C, main text), in conjunction with its disordered conformation, prevents large chemical shift perturbations. Similar experiments are not feasible in ALIX due to its large size (~100 kDa), which leads to resonance line-broadening.

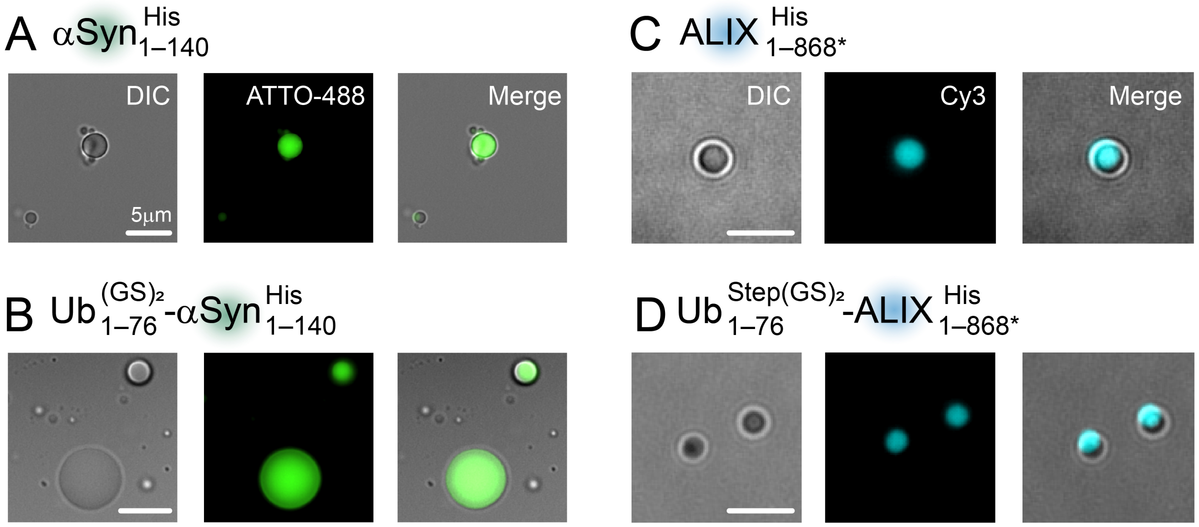

Figure S8. Phase separation of α-synuclein and ALIX and the impact of monoubiquitination. Representative microscopy images of condensates formed by ATTO-488 labeled (A) αSyn^{His}_{1-140} and (B) its monoubiquitinated counterpart, and Cy3-labeled (C) $ALIX^{His}_{1-868*}$ and (D) its monoubiquitinated species. NEDD4L and WWP2 were used for monoubiquitination of α-synuclein and ALIX, respectively. Phase separation experiments for α-synuclein were performed at room temperature in 25 mM Tris, pH 7.4, 50 mM NaCl, 1 mM EDTA and 10% (w/v) PEG-8000, with 200 μM protein. For ALIX, experiments were performed at room temperature in 25 mM HEPES, pH 7.4, 150 mM NaCl, 1 mM DTT, 1 mM EDTA, and 5% (w/v) PEG-4000, with 50 μM protein. The type and concentration of crowding agents, i.e., PEG-8000 and PEG-4000, were selected based on prior reports (Elias et al., manuscript submitted and Ray et al., Nat. Chem. 2020, respectively).[17, 23] The lower limits of their concentrations were 3% and 4% for PEG-8000 and PEG-4000, respectively, which created fewer and smaller condensates of αSyn^{His}_{1-140} and $ALIX^{His}_{1-868*}$. All fluorescently labeled droplets were prepared using 10% labeled and 90% unlabeled protein. Images were taken immediately after the formation of condensates.

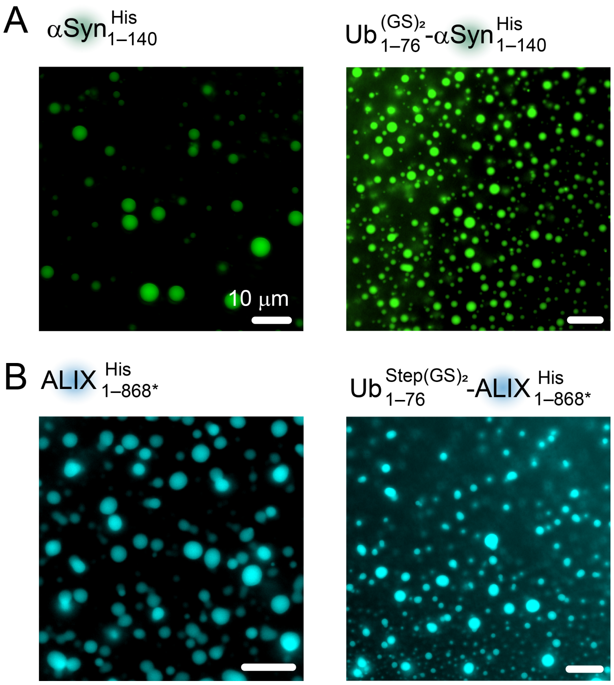

Figure S9. Panoramic images showing the phase separation of proteins used in current study. Representative fluorescence microscopy images showing condensates of (**A**) ATTO488-labeled $\alpha\text{Syn}^{\text{His}}_{1\text{–}140}$ and its NEDD4L-mediated monoubiquitinated counterpart and (**B**) Cy3-labeled $\text{ALIX}^{\text{His}}_{1\text{–}868*}$ and its WWP2-mediated monoubiquitinated counterpart. Experimental and buffer conditions were the same as described in Fig. S8 caption.

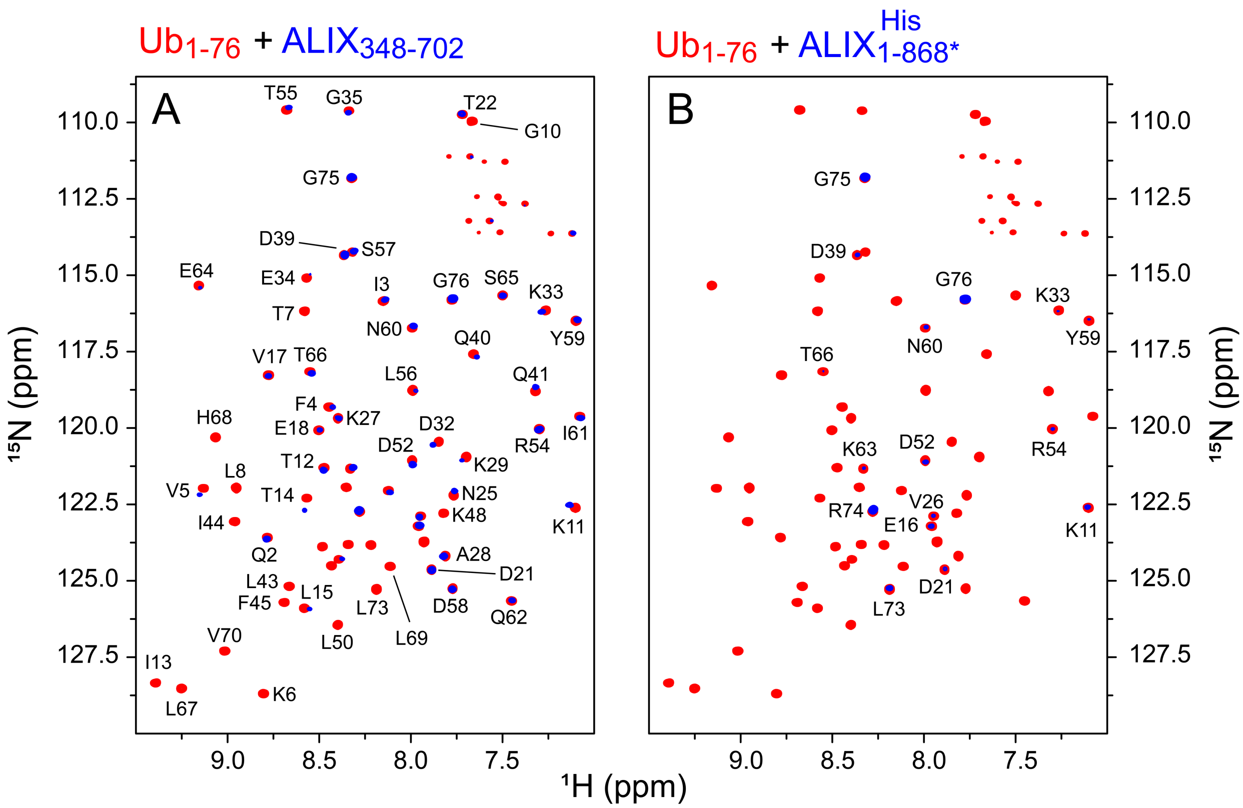

Figure S10. NMR analyses of ubiquitin–ALIX interactions. Expanded regions of ^{1}H-^{15}N TROSY-HSQC spectra of 100 μM ^{15}N-labeled $Ub_{1\text{-}76}$, in the absence (red) and presence (blue) of its unlabeled binding partners (100 μM each), namely (**A**) the V-domain of ALIX, $ALIX_{348\text{–}702}$, and (**B**) full-length ALIX, $ALIX^{His}_{1\text{–}868*}$. A few isolated ^{1}H–^{15}N cross-peaks are labeled. All spectra were recorded at a spectrometer ^{1}H frequency of 800 MHz. The experimental conditions were as follows: 20 mM sodium phosphate, pH 6.5, 1 mM EDTA, and 1 mM TCEP at 27 °C.

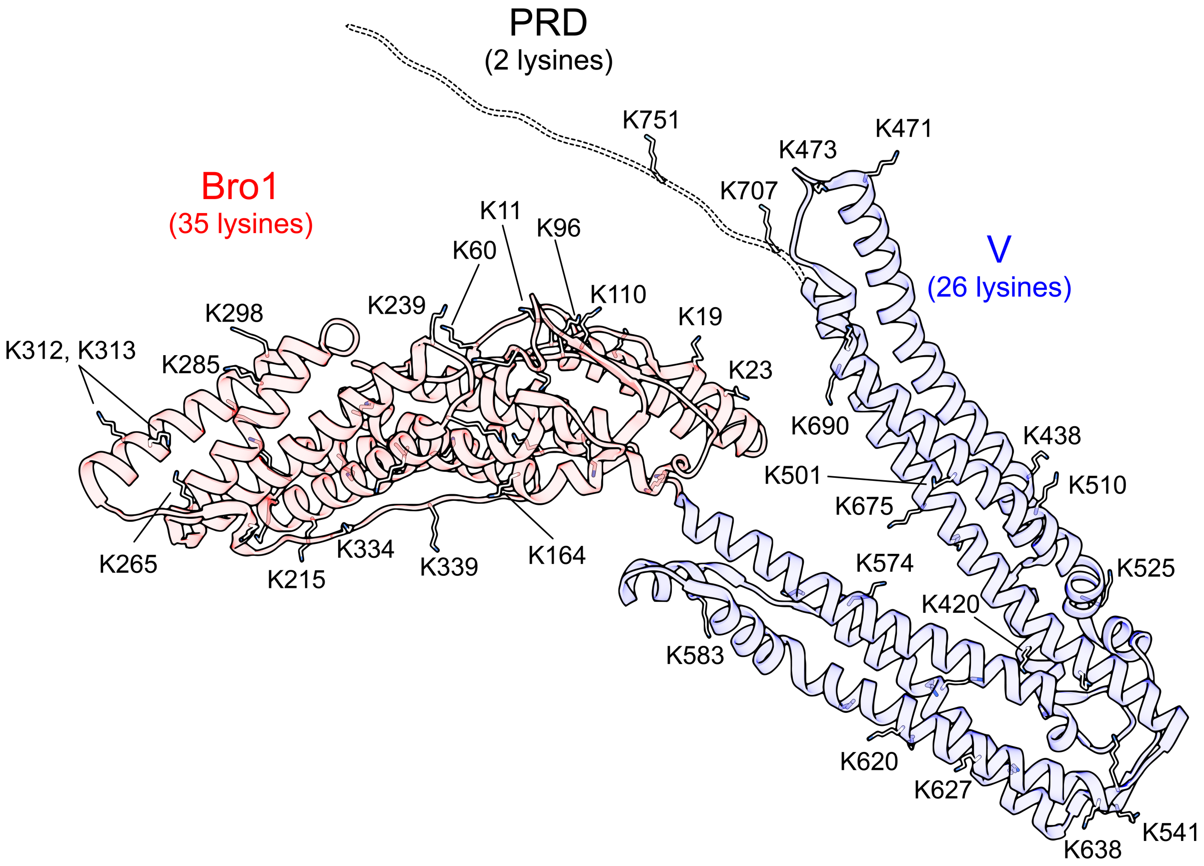

Figure S11. ALIX and the location of its lysine residues. Schematic of ALIX comprising Bro1, V, and PRD (red and blue ribbons and dashed black lines, respectively), derived from the X-ray structure of Bro1-V domains (PDB entry: 2XS1).[24] Note that unlike Bro1 and V domains, the structure of PRD is not available as it is disordered.[1] The lysine residues of ALIX are shown in stick representation. A few of the isolated residues are labeled.

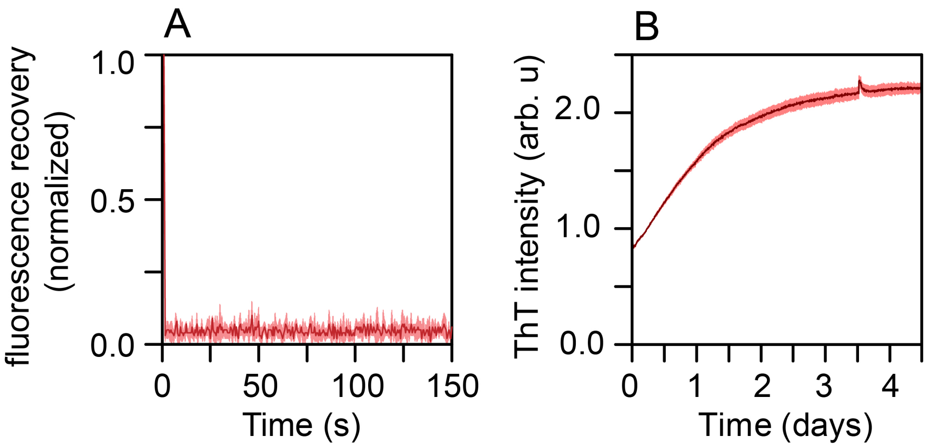

Figure S12. Aggregation properties of NEDD4L-mediated monoubiquitinated ALIX. (**A**) Lack of FRAP recoveries for the freshly prepared condensates of NEDD4L-mediated monoubiquitinated $ALIX^{His}_{1-868*}$ (red), n = 3, solid line (mean) and shaded region (SD). (**B**) Aggregation of NEDD4L-mediated monoubiquitinated $ALIX^{His}_{1-868*}$ studied by ThT assays, n = 2.

Table S1. Recombinant constructs used in current study.

Construct	UniProt entry	Addgene Accession no.	Competent cells[(a)]	Induction temperature[(b)]	Growth medium[(c)]	Yield (mg/1L)[(d)]
UbE1	P22314	186804	BL21-AI	16 °C	TB	10 mg
UBE2D3	P61077	186805	BL21-AI	16 °C	TB	100 mg
NEDD4L	Q96PU5	186806	BL21-AI	16 °C	TB	20 mg
WWP2	O00308	186807	BL21-AI	16 °C	TB	15 mg
$Ub_{1\text{-}76}^{Strep(GS)_2}$	P0CG48	186803	BL21(DE3)	16 °C	LB	12 mg
$\alpha Syn_{1\text{-}140}^{His}$	P37840	186802	BL21-AI	37 °C	LB	20 mg
$ALIX_{1\text{-}868*}^{His}$	Q8WUM4	186808	BL21-AI	16 °C	LB	30 mg
$ALIX_{348–702}$	Q8WUM4	189819	BL21-(DE3)	16 °C	LB	40 mg

a) BL21(DE3) and BL21-AI cells were obtained from Agilent (catalog no. 200131) and Thermo Fisher Scientific (catalog no. C607003), respectively.

b) Cultures were grown overnight upon induction with 1 mM IPTG (and 0.2% w/v arabinose; the latter was used for BL21-AI cells).

c) TB broth and LB capsules were obtained from Thermo Fischer Scientific (catalog no. BP9728-500) and MP Biomedicals (catalog no. 3002-036), respectively, and were used according to the manufacturers' protocols.

d) The yield represents total protein obtained from a liter of bacterial culture. In the case of ubiquitinating enzymes, $Ub_{1–76}^{Strep(GS)_2}$, and $ALIX_{348–702}$, it represents the total amount of protein obtained after cleaving off the N-terminal purification tag by TEV protease.

Table S2. Components of in vitro ubiquitination reactions and the yields of corresponding monoubiquitinated products[(a,b,c)].

Reaction Component	$\alpha Syn_{1\text{-}140}^{His}$ (250 μM)	$ALIX_{1\text{-}868*}^{His}$ (150 μM)	$ALIX_{1\text{-}868*}^{His}$ (150 μM)
UbE1	15 μM	15 μM	15 μM
UBE2D3	20 μM	20 μM	20 μM
NEDD4L	25 μM	—	25 μM
WWP2	—	25 μM	—
$Ub_{1\text{-}76}^{Strep(GS)_2}$	500 μM	500 μM	500 μM
Monoubiquitinated product	2.5 mg / 5 mL	1.3 mg / 4 mL	1.2 mg / 4 mL

a) All reaction components were mixed, and the corresponding mixture was dialyzed against the buffer containing 50 mM Tris, pH 7.5, 1 mM DTT, 1 mM $MgCl_2$, and 1.5 mM ATP for 5 h at 30 °C.

b) In vitro ubiquitination reaction of αSyn_{1-140}^{His} + WWP2 was not performed as unlike ALIX, WWP2 has not been shown to ubiquitinate α-synuclein in vivo.

c) Unmodified target proteins, i.e., the flow-through fractions from Step-3 (see Fig. 1A, main text), can be reused in the next round of in vitro ubiquitination reactions.

Table S3. Quantification of the site-specific frequency of monoubiquitination for $\alpha Syn^{His}_{1\text{-}140}$ and $ALIX^{His}_{1\text{-}868*}$ using a chemical proteomics approach[(a,b)].

Lysine Residues	NEDD4L Average percent stoichiometry	WWP2 Average percent stoichiometry
$\alpha Syn^{His}_{1\text{-}140}$ [(c)]		
K6/K10/K12	19.2 ± 0.5	—
K21/K23	11.3 ± 2.4	—
K32/K34	0.2 ± 0.2	—
K43/K45	5.0 ± 1.2	—
K58/K60	1.2 ± 1.2	—
K80	20.7 ± 2.4	—
K96/K97/K102	42.4 ± 0.2	—
$ALIX^{His}_{1\text{-}868*}$ [(d)]		
K10/K11	0.1 ± 0.0	0.7 ± 0.6
K19/K23	1.6 ± 1.1	1.5 ± 0.4
K48	0.3 ± 0.1	0.2 ± 0.1
K60	0.9 ± 0.3	2.1 ± 0.5
K81	—	—
K96	—	—
K101	—	—
K110	—	—
K120	—	—
K147	—	—
K151	—	—
K164	—	—
K202	0.2 ± 0.0	0.2 ± 0.1

Table S3 (cont'd).

Lysine Residues	NEDD4L Average percent stoichiometry	WWP2 Average percent stoichiometry
K207	—	—
K209	—	—
K215	—	—
K229	—	—
K234	—	—
K239	—	—
K248	3.2 ± 0.5	4.6 ± 1.0
K265	1.7 ± 0.2	3.9 ± 0.5
K268/K269	1.0 ± 0.1	0.9 ± 0.1
K285	0.9 ± 0.2	0.9 ± 0.0
K298/K303	0.8 ± 0.5	1.2 ± 0.2
K312/K313	0.3 ± 0.2	0.4 ± 0.0
K327	—	—
K334	—	—
K339	—	—
K350	—	—
K357	0.6 ± 0.2	0.6 ± 0.1
K374	0.2 ± 0.1	0.3 ± 0.1
K420	7.6 ± 7.6	23.7± 1.1
K438	2.4 ± 0.3	3.4 ± 0.3
K471/K473	1.9 ± 0.2	3.6 ± 0.1
K486	0.5 ± 0.4	0.6 ± 0.1
K501/K510	42.4 ± 6.7	8.6 ± 2.7
K525	2.8 ± 1.3	1.2 ± 0.5
K541	1.8 ± 1.8	0.6 ± 0.4

Table S3 (cont'd).

	NEDD4L	WWP2
Lysine Residues	Average percent stoichiometry	Average percent stoichiometry
K553/K563/K564	0.3 ± 0.1	1.1 ± 0.9
K574	—	—
K583	0.0 ± 0.0	0.0 ± 0.0
K614	0.3 ± 0.0	0.4 ± 0.0
K620/K621	0.0 ± 0.0	0.0 ± 0.0
K627	0.9 ± 0.2	1.5 ± 0.6
K638/K640	7.4 ± 1.2	11.1 ± 3.4
K654	1.5 ± 1.5	1.7 ± 1.7
K671	0.8 ± 0.6	0.3 ± 0.2
K675	0.0 ± 0.0	0.2 ± 0.2
K690	1.2 ± 0.1	1.3 ± 0.2
K699/K707	5.9 ± 1.2	6.4 ± 0.6
K751	10.3 ± 0.7	16.8 ± 0.5

a) Stoichiometry for each site was determined by dividing the precursor ion LC-MS chromatographic peak area of a monoubiquitinated peptide by the sum of the peak areas of the peptide's mono- and non-ubiquitinated forms. Percentages for each site were calculated by summing the stoichiometries of each individual reaction (n = 2), which represented the total pool of monoubiquitinated species. The reported values represent average percentages of these two independent reactions and the corresponding standard deviations.

b) In vitro ubiquitination reaction of $\alpha\mathrm{Syn}^{\mathrm{His}}_{1\text{–}140}$ + WWP2 was not performed as unlike ALIX, WWP2 has not been shown to ubiquitinate α-synuclein in vivo.

c) The average percentages for the MBR (i.e., residues 6, 10, 12, 21, 23, 32, 34, 43, 45, 58 and 60) are 36.9 ± 3.0%.

d) The average percentages for individual ALIX domains are as follows: Bro1 = 11.5 ± 1.4%, V = 72.2 ± 10.6%, and PRD = 16.2 ± 1.4% for NEDD4L, and Bro1 = 17.2 ± 1.4%, V = 59.6 ± 5.0%, and PRD = 23.2 ± 0.8% for WWP2, respectively.

Table S4. Quantification of the site-specific frequency of multi-mono/polyubiquitinated $\alpha Syn^{His}_{1\text{-}140}$ using chemical proteomics approach[(a)].

	Multi-mono/polyubiquitinated $\alpha Syn^{His}_{1\text{-}140}$	Fold-change[(b)]
Lysine Residues	Average percent stoichiometry	Multi-mono/poly vs. monoubiquitinated $\alpha Syn^{His}_{1\text{-}140}$
$\alpha Syn^{His}_{1\text{-}140}$ [(c)]		
K6/K10/K12	21.7 ± 0.2	1.1
K21/K23	7.8 ± 0.8	0.7
K32/K34	3.5 ± 0.1	17.5
K43/K45	13.5 ± 0.1	2.7
K58/K60	10.7 ± 2.4	8.9
K80	12.1 ± 0.8	0.6
K96/K97/K102	30.7 ± 2.6	0.7

a) Samples from lanes 8–9 (cf. Fig. S4B) were subjected to quantitative chemical proteomics. The reported values represent average percentages of two independent reactions and the corresponding standard deviations.

b) The fold-change represents the corresponding change between multi-mono/poly vs. mono-ubiquitinated $\alpha Syn^{His}_{1\text{-}140}$ (cf. Table S3).

c) The average percentages for the MBR (i.e., residues 6, 10, 12, 21, 23, 32, 34, 43, 45, 58 and 60) are 57.3 ± 2.5%.

References

(1) Elias, R. D.; Ma, W.; Ghirlando, R.; Schwieters, C. D.; Reddy, V. S.; Deshmukh, L., Proline-rich domain of human ALIX contains multiple TSG101-UEV interaction sites and forms phosphorylation-mediated reversible amyloids. *Proc. Natl. Acad. Sci. U.S.A.* **2020,** *117* (39), 24274-24284.

(2) Elias, R. D.; Ramaraju, B.; Deshmukh, L., Mechanistic roles of tyrosine phosphorylation in reversible amyloids, autoinhibition, and endosomal membrane association of ALIX. *J. Biol. Chem.* **2021,** *297* (5), 101328.

(3) Ramaraju, B.; Nelson, S. L.; Zheng, W.; Ghirlando, R.; Deshmukh, L., Quantitative NMR study of insulin-degrading enzyme using amyloid-beta and HIV-1 p6 elucidates its chaperone activity. *Biochemistry* **2021,** *60* (33), 2519-2523.

(4) Huth, J. R.; Bewley, C. A.; Jackson, B. M.; Hinnebusch, A. G.; Clore, G. M.; Gronenborn, A. M., Design of an expression system for detecting folded protein domains and mapping macromolecular interactions by NMR. *Protein Sci.* **1997,** *6* (11), 2359-64.

(5) Li, Y.; Evers, J.; Luo, A.; Erber, L.; Postler, Z.; Chen, Y., A quantitative chemical proteomics approach for site-specific stoichiometry analysis of ubiquitination. *Angew. Chem., Int. Ed. Engl.* **2019,** *58* (2), 537-541.

(6) Cox, J.; Mann, M., MaxQuant enables high peptide identification rates, individualized p.p.b.-range mass accuracies and proteome-wide protein quantification. *Nat. Biotechnol.* **2008,** *26* (12), 1367-72.

(7) Rückert, M.; Otting, G., Alignment of biological macromolecules in novel nonionic liquid crystalline media for NMR experiments. *J. Am. Chem. Soc.* **2000,** *122* (32), 7793-7797.

(8) Delaglio, F.; Grzesiek, S.; Vuister, G. W.; Zhu, G.; Pfeifer, J.; Bax, A., NMRPipe: A multidimensional spectral processing system based on UNIX pipes. *J. Biomol. NMR* **1995,** *6* (3), 277-293.

(9) Vranken, W. F.; Boucher, W.; Stevens, T. J.; Fogh, R. H.; Pajon, A.; Llinas, M.; Ulrich, E. L.; Markley, J. L.; Ionides, J.; Laue, E. D., The CCPN data model for NMR spectroscopy: Development of a software pipeline. *Proteins: Struct. Funct. Genet.* **2005,** *59* (4), 687-696.

(10) Clore, G. M.; Gronenborn, A. M., Determining the structures of large proteins and protein complexes by NMR. *Trends Biotechnol* **1998,** *16* (1), 22-34.

(11) Fitzkee, N. C.; Bax, A., Facile measurement of ^{1}H-^{15}N residual dipolar couplings in larger perdeuterated proteins. *J. Biomol. NMR* **2010,** *48* (2), 65-70.

(12) Schwieters, C. D.; Bermejo, G. A.; Clore, G. M., Xplor-NIH for molecular structure determination from NMR and other data sources. *Protein Sci* **2018,** *27* (1), 26-40.

(13) Gidi, Y.; Bayram, S.; Ablenas, C. J.; Blum, A. S.; Cosa, G., Efficient one-step PEG-silane passivation of glass surfaces for single-molecule fluorescence studies. *ACS Appl. Mater. Interfaces* **2018,** *10* (46), 39505-39511.

(14) Paravastu, A. K.; Qahwash, I.; Leapman, R. D.; Meredith, S. C.; Tycko, R., Seeded growth of β-amyloid fibrils from Alzheimer's brain-derived fibrils produces a distinct fibril structure. *Proc. Natl. Acad. Sci. U.S.A.* **2009,** *106* (18), 7443-7448.

(15) Deshmukh, L.; Ghirlando, R.; Clore, G. M., Conformation and dynamics of the Gag polyprotein of the human immunodeficiency virus 1 studied by NMR spectroscopy. *Proc. Natl. Acad. Sci. U.S.A.* **2015,** *112* (11), 3374-9.

(16) Schmidt, T. G. M.; Batz, L.; Bonet, L.; Carl, U.; Holzapfel, G.; Kiem, K.; Matulewicz, K.; Niermeier, D.; Schuchardt, I.; Stanar, K., Development of the Twin-Strep-tag and its application

for purification of recombinant proteins from cell culture supernatants. *Protein Expr. Purif.* **2013,** *92* (1), 54-61.
(17) Elias, R. D.; Zhu, Y.; Su, Q.; Ghirlando, R.; Zhang, J.; Deshmukh, L., Reversible phase separation of ESCRT-protein ALIX through tyrosine phosphorylation. **submitted**.
(18) Vijay-Kumar, S.; Bugg, C. E.; Cook, W. J., Structure of ubiquitin refined at 1.8 A resolution. *J. Mol. Biol.* **1987,** *194* (3), 531-44.
(19) Clore, G. M.; Garrett, D. S., R-factor, free R, and complete cross-validation for dipolar coupling refinement of NMR structures. *J. Am. Chem. Soc.* **1999,** *121* (39), 9008-9012.
(20) Cytiva.com. Strep-Tactin XT Sepharose affinity chromatography resin, StrepTrap XT prepacked columns. https://cdn.cytivalifesciences.com/api/public/content/digi-32505-pdf.
(21) Köhler, M.; Karner, A.; Leitner, M.; Hytönen, V. P.; Kulomaa, M.; Hinterdorfer, P.; Ebner, A., pH-dependent deformations of the energy landscape of avidin-like proteins investigated by single molecule force spectroscopy. *Molecules* **2014,** *19* (8), 12531-46.
(22) Geoghegan, K. F.; Dixon, H. B.; Rosner, P. J.; Hoth, L. R.; Lanzetti, A. J.; Borzilleri, K. A.; Marr, E. S.; Pezzullo, L. H.; Martin, L. B.; LeMotte, P. K.; McColl, A. S.; Kamath, A. V.; Stroh, J. G., Spontaneous alpha-N-6-phosphogluconoylation of a "His tag" in Escherichia coli: the cause of extra mass of 258 or 178 Da in fusion proteins. *Anal. Biochem.* **1999,** *267* (1), 169-84.
(23) Ray, S.; Singh, N.; Kumar, R.; Patel, K.; Pandey, S.; Datta, D.; Mahato, J.; Panigrahi, R.; Navalkar, A.; Mehra, S.; Gadhe, L.; Chatterjee, D.; Sawner, A. S.; Maiti, S.; Bhatia, S.; Gerez, J. A.; Chowdhury, A.; Kumar, A.; Padinhateeri, R.; Riek, R.; Krishnamoorthy, G.; Maji, S. K., α-Synuclein aggregation nucleates through liquid-liquid phase separation. *Nat. Chem.* **2020,** *12* (8), 705-716.
(24) Zhai, Q.; Landesman, M. B.; Robinson, H.; Sundquist, W. I.; Hill, C. P., Identification and structural characterization of the ALIX-binding late domains of simian immunodeficiency virus SIVmac239 and SIVagmTan-1. *J Virol* **2011,** *85* (1), 632-7.
